# Evaluation of the Success of High-Throughput Physiologically Based Pharmacokinetic (HT-PBPK) Modeling Predictions to Inform Early Drug Discovery

**DOI:** 10.1021/acs.molpharmaceut.2c00040

**Published:** 2022-04-27

**Authors:** Doha Naga, Neil Parrott, Gerhard F. Ecker, Andrés Olivares-Morales

**Affiliations:** † Roche Pharma Research and Early Development (pRED), Roche Innovation Center Basel, Grenzacherstrasse 124, 4070 Basel, Switzerland; ‡ Department of Pharmaceutical Sciences, University of Vienna, 1090 Vienna, Austria

**Keywords:** drug discovery, PBPK models, HT-PBPK, physicochemical properties, clearance predictions and machine learning

## Abstract

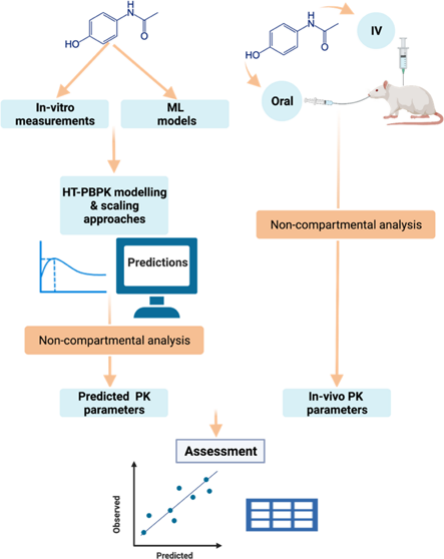

Minimizing in vitro and in vivo testing in early drug discovery with the use of physiologically based pharmacokinetic (PBPK) modeling and machine learning (ML) approaches has the potential to reduce discovery cycle times and animal experimentation. However, the prediction success of such an approach has not been shown for a larger and diverse set of compounds representative of a lead optimization pipeline. In this study, the prediction success of the oral (PO) and intravenous (IV) pharmacokinetics (PK) parameters in rats was assessed using a “bottom-up” approach, combining in vitro and ML inputs with a PBPK model. More than 240 compounds for which all of the necessary inputs and PK data were available were used for this assessment. Different clearance scaling approaches were assessed, using hepatocyte intrinsic clearance and protein binding as inputs. In addition, a novel high-throughput PBPK (HT-PBPK) approach was evaluated to assess the scalability of PBPK predictions for a larger number of compounds in drug discovery. The results showed that bottom-up PBPK modeling was able to predict the rat IV and PO PK parameters for the majority of compounds within a 2- to 3-fold error range, using both direct scaling and dilution methods for clearance predictions. The use of only ML-predicted inputs from the structure did not perform well when using in vitro inputs, likely due to clearance miss predictions. The HT-PBPK approach produced comparable results to the full PBPK modeling approach but reduced the simulation time from hours to seconds. In conclusion, a bottom-up PBPK and HT-PBPK approach can successfully predict the PK parameters and guide early discovery by informing compound prioritization, provided that good in vitro assays are in place for key parameters such as clearance.

## Introduction

Absorption, distribution, metabolism, and elimination (ADME) and pharmacokinetics (PK) in general play a key role in drug discovery and compound optimization.^1,2^ The ADME process depends on the interplay of the compound’s physicochemical properties, the route of administration, and the physiologically related parameters of the species to which the drug is administered (e.g., intestinal transit times, tissue composition, blood flow, metabolizing enzymes).^3−5^

Assessment of the PK properties is an integral part of drug development and is usually conducted as a part of the lead identification/optimization (LI/LO) process through in vitro assays followed by in vivo studies prior to clinical testing.^6,7^ These assays and studies are performed to select and prioritize compounds according to their ADME and pharmacokinetic properties.^4^ They are also necessary to ensure the selection of a drug candidate with the potential for a favorable human PK to progress into phase 0 and subsequently into human studies (phase 1 onwards), although the direct transfer of human pharmacokinetics properties such as bioavailability from nonclinical species has limited value.^8^

In vivo studies, however, are labor-intensive, time-consuming, and require animal experimentation.^9^ In silico alternatives to such studies are highly encouraged to reduce cycle times and minimize animal experimentation according to the 3R principles (replacement, reduction, and refinement).^10^ Therefore, the prediction of key PK properties directly from structure using in silico methods or minimal in vitro data could support early compound drug design strategies and help discovery scientists to select the best candidates for further progression.

By integrating system-dependent and compound-dependent parameters, physiologically based pharmacokinetic (PBPK) models can be used in early discovery to predict the PK of new drug candidates.^11^ PBPK models describe the fate of a drug using detailed mathematical equations to describe a multicompartmental system with compartments representing organ and tissue volumes and linked by rates based on blood flows. When PBPK models are combined with in vitro to in vivo extrapolation (IVIVE), they are a powerful tool for the understanding and prediction of pharmacokinetics. A key task for preclinical drug discovery is the selection of molecules with human pharmacokinetics, which, when combined with pharmacodynamic measures, allow a reasonable therapeutic dosing regimen. Previous studies have shown that PBPK modeling can provide an optimal basis for the prediction of clinical pharmacokinetics.^12^ This is achieved through the integration of in vitro data predictive of key pharmacokinetic processes into a realistic physiological framework. If highly predictive in vitro data were available for all relevant processes, then a direct prediction of human pharmacokinetics would be possible. However, in practice, relevant assays are limited in their predictive accuracy. Therefore, one strategy is to improve the success of human PBPK predictions by including a verification step for the model predictions using in vivo pharmacokinetics data obtained in nonclinical species and further refine the model if necessary, by applying the learnings in one species to inform the human PBPK predictions.^13,14^

However, the application of PBPK models in the early discovery space for medicinal chemistry optimization cycles prior to clinical candidate selection is currently limited.^15,16^ In the early discovery space, other tools such as QSAR, machine learning (ML) models, and/or simple early human dose calculations using spreadsheets combined with IVIVE are generally preferred due to their scalability and ease of use. However, such tools do not provide a holistic picture of the interplay that the different parameters can have on human PK. For example, while systemic clearance (CL) and *V*_ss_ might be readily estimated using mechanistic equations such as the well-stirred model for hepatic clearance and the tissue-composition-based models which estimate tissue to plasma partition coefficients and *V*_ss_,^17−19^ no simple approach exists that allows the estimation of the rate and extent of oral absorption and bioavailability based on intrinsic in vitro and/or in silico inputs. The complex interplay between release, dissolution, permeation, and first-pass metabolism in oral absorption requires complex models such as those in the well-known PBPK models, such as the Advanced Compartmental Absorption and Transit (ACAT) model in GastroPlus or the advanced dissolution, absorption, and metabolism (ADAM) model in SimCYP.^20,21^ Furthermore, PBPK models provide the advantage of allowing sensitivity analyses to assess the impact that the input parameters might have on the overall PK profile of a compound, which cannot be assessed when using simple correlations and extrapolation of PK from nonclinical species.^22,23^ Another advantage of applying early discovery PBPK is the continuity with the PBPK modeling approaches, which are already well established at the later stages of drug discovery and clinical development.

Factors likely to have limited the use of PBPK models in the early stages of discovery are the scarcity of data available to feed into the models and a lack of confidence in bottom-up PBPK modeling. Efforts to demonstrate the prediction success of bottom-up PBPK models have been carried out by large consortia of academic and industrial collaborators, for example, the IMI Oral Bio Pharmaceutics tools (OrBiTo) project^24−26^ and the PhRMA CPCDC initiative.^27−29^ These initiatives highlighted some of the challenges and limitations of early bottom-up PBPK predictions, which include the performance of clearance predictions using in vitro systems, such as hepatocytes, where a trend to underestimation has been observed.^30−32^ However, these consortia focused mainly on human predictions where significant amounts of data were available, and might not reflect the situation in early drug discovery. A few literature examples have reported on the prediction success of PBPK models in early discovery. To assess the potential to guide compound optimization, Parrott and co-workers^33^ evaluated the utility of PBPK models in rat to predict in vivo PK of 68 chemically diverse compounds. Using a mixture of in vitro measured and in silico predicted properties, they were able to predict rat PK parameters with reasonable precision and estimated that the approach could be valuable to prioritize and rank compounds in early projects. More recently, Daga and co-workers investigated the amalgamation of machine learning models with PBPK to predict bioavailability and inform compound optimization within chemical classes.^34^ Using a structure-based model trained against a fitted clearance and integrated into a PBPK model, they demonstrated good prediction of oral bioavailability for three distinct compound series. While these models could be highly beneficial to inform medicinal chemistry efforts in advance of synthesis, the applicability domain might be limited to the specific chemical space of each series.

Herein, we further evaluate whether fully bottom-up high-throughput (HT) PBPK predictions, combining in vitro and in silico inputs, can be used to inform drug design and early drug discovery. We have assessed the prediction success for both oral (PO) and intravenous (IV) PK parameters in rats for a library of more than 240 structurally diverse compounds using representative data from the Roche Pharma Research and Early Development (pRED) discovery pipeline. In addition, we have assessed the prediction performance of PBPK models using input parameters predicted from the structure with commercially available machine learning models. The final aim is to establish the basis for a framework that enables use of HT-PBPK modeling in early discovery.

## Materials and Methods

### Data Retrieval and Curation

In-house databases were screened for all compounds with pharmacokinetic studies after single-dose intravenous (IV) and oral administration (PO) in rats and with the measured in vitro data necessary to perform PBPK simulations. All of the studies had the PK parameters of interest for this assessment, which were: plasma clearance (CL), volume of distribution at steady state (*V*_ss_), area under the concentration versus time curve from zero to infinity (AUC_inf_), the maximal concentration after single-dose administration (*C*_max_), and the oral bioavailability (*F*_oral_). The data were checked for quality and consistency. In addition, to be representative of early discovery PK studies (i.e., first in animal) instead of more mechanistic studies such as formulation development or safety studies, the search for oral PK experiments was limited to oral doses of less than 50 mg/kg. The rat PK studies were performed in at least two male rats (Wistar, Sprague-Dawley, or Fischer 344) per experiment with compounds administered as a bolus for the IV route or via gavage for PO. Formulations were a solution (IV or PO) or micro-suspension (PO only), and the doses ranged from 0.03 to 10 mg/kg for IV experiments and from 0.2 to 34 mg/kg for PO. Serial blood samples were taken for up to 48 h post dose using either a catheter or serial tail vein microsampling. The plasma samples were subsequently analyzed and quantified for the administered compound using liquid chromatography with tandem mass spectrometry (LC-MS/MS). Noncompartmental analysis (NCA) was used to determine PK parameters for each animal, which were then presented as the arithmetic mean for each study arm (route of administration, experiment identifier, and dose).

The required measured drug-specific properties for PBPK modeling were those considered to represent the minimal set of input data needed^11,33^ and were defined as: octanol/water partition coefficient (Log *D*), aqueous solubility (thermodynamic or kinetic), passive cellular permeability measured in Lilly Laboratories Cell Porcine Kidney 1 (LLC-PK1) cells, metabolic stability measured as intrinsic clearance in suspension hepatocytes (CL_int,he’s_), and plasma protein binding (*f*_up_). Briefly, the in vitro measurements were performed as follows: Log *D* values at a defined pH (in general 7.4) were measured in a high-throughput assay derived from the conventional shake-flask method.^35^ The fraction unbound in rat plasma was measured with equilibrium dialysis at 1 μM. The aqueous solubility was measured in a high-throughput lyophilization assay (LYSA)^36^ using 10 mM dimethyl sulfoxide (DMSO) stock solution and a phosphate buffer at pH 6.5. In vitro values for solubility in fed and fasted state simulated intestinal fluids (FaSSIF and FeSSIF, respectively) were used when available (132 compounds) and were measured using the conventional shake-flask method.^35^ Passive permeability in LLC-PK1 cells overexpressing P-glycoprotein (P-gp) was measured at 1 μM, and the intrinsic clearance in cryopreserved suspended rat hepatocytes was measured by substrate depletion at 1 μM. Further details of the permeability and hepatocyte stability assay can be found elsewhere.^37^ The measured passive permeability in LLC-PK1 cells was translated to human intestinal effective permeability (*P*_eff_) using an in-house correlation based on measurements for reference drugs with known jejunal *P*_eff_ (Log 10(*P*_eff_) = 0.607 Log 10(*P*_app,LLC-PK1_) + 2.014). Rat *P*_eff_ was then estimated from human *P*_eff_ using a correlation within GastroPlus (*P*_eff_rat_ = 1.14 × *P*_eff_man_). When in vitro values were not available, predicted parameters were substituted by ML predicted values, particularly for rat blood-to-plasma partitioning ratio (BP), FaSSIF and FeSSIF solubility. Also since the ML models for ionization state and p*K*_a_ value were considered highly reliable,^38^ these were used for all compounds. All of the aforementioned parameters were predicted from structure using the ADMET predictor (AP) software version 10.1 (Simulations Plus, Lancaster, CA).

### Compound Classification

To identify relationships between compound classes and prediction accuracy, compounds were classified according to several criteria, namely, chemotype, ionization, in vivo systemic clearance, extent of plasma protein binding, and Extended Clearance Classification System (ECCS).^39^ Further details are given below.

### Chemotype

Compound structural classes were generated using the MedChem Studio module in ADMET predictor version 10.1 with two methods. (A) The ring-anchored system that generates classes with scaffolds based on ring systems (single and fused) as well as those connected by non-ring linker atoms. (B) The (fingerprint clustering) option, selecting extended connectivity fingerprint (ECFP)^40^ as descriptors and 0.4 (default) as a minimum Tanimoto similarity^41−43^ in the clustering options. The option to “generate maximum common substructures” was also enabled to increase the size of each individual cluster.

### Ionization

Ionization state of the molecules at pH 7.4 was computed using four ionization descriptors in ADMET predictor. These descriptors estimate the cumulative contributions of (i) purely anionic species (FAnion), (ii) purely cationic species (FCation), (iii) fraction unionized (FUnion) at physiological pH (7.4), and (iv) the fraction zwitterionic (FZwitter). Compounds were then categorized into acidic (FAnion > 0.5), basic (FCation > 0.5), neutral (FUnion > 0.5 and Fzwitter FZwitter < 0.5), and zwitterions (FUnion > 0.5 and FZwitter ≥ 0.5).

### Systemic Clearance

Compounds were split into four in vivo blood clearance categories according to the estimated hepatic extraction ratio, calculated assuming a rat liver blood flow of 60 mL/min/kg. The five categories were: very low: <6, low: 6–18, moderate: 18–42, and high: 42–60 mL/min/kg.

### Extent of Protein Binding

Two categories were defined: highly bound, where the *f*_up_ in rats is less than 2%, and moderately bound, where *f*_up_ is greater than or equal to 2%

### Extended Clearance Classification System (ECCS)

The ECCS system predicts the main route of drug clearance based on passive membrane permeability (*P*_app_) (high when *P*_app_ ≥ 5 × 10^–6^ cm/s and low when *P*_app_ ≤ 5 × 10^–6^ cm/s), ionization state (acids and zwitterions vs bases and neutrals), and molecular weight (above or below 400 g/mol). Accordingly, the ECCS classes are identified as follows: class 1a and class 2 (metabolic clearance), class 1b (hepatic uptake), class 3a and class 4 (renal clearance), and finally class 3b (transporter-mediated hepatic uptake or renal clearance).^39^ In this study, the ECCS classification was predicted in silico using ADMET predictor v 10.1, which assigns the class according to its own ionization and permeability models. The ionization state is given by the four aforementioned ionization descriptors (FAnion, FCation, FZwitter, FUnion) and the permeability class is predicted using an artificial neural network ensemble (ANNE) model trained on Madin–Darby Canine Kidney-Limited Efflux cell line (MDCK-LE) permeability built from the data used by Varma et al.^39^

### IVIVE of Clearance

The plasma clearance was scaled from unbound intrinsic clearance using GastroPlus version 9.8 or ADMET predictor 10.1 (Simulations Plus, Lancaster, CA) based on values measured by substrate depletion in cultures of suspended rat hepatocytes (CL_int,heps(u)_). The measured CL_int,heps_ was corrected for nonspecific binding using eq 1, where fu_inc_ is the fraction unbound in the incubation.
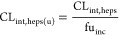
1

Four different clearance scaling approaches were used based on different assumptions with regard to the estimation of fu_inc_:

(a) Direct scaling, where fu_inc_ is assumed to equal *f*_up_(44,45)
(b) Dilution method: fu_inc_ is calculated based on eq 2
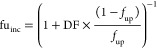
2where *f*_up_ is the fraction unbound in plasma in rats and DF is the dilution factor. This method is similar to the direct scaling; however, it takes into account the dilution factor between the measured *f*_up_ and the level of plasma proteins in the incubation media (in this case, DF = 1/10 since 10% bovine serum albumin [BSA] is added to the hepatocyte incubation). Further details are described in the work of Berezhkovskiy et al.^46^
(c) Unbound: assumes that measured intrinsic clearance is unbound (fu_inc_ = 1) and
(d) In silico Austin method (or default in silico method in ADMET predictor and GastroPlus), where fu_inc_ was predicted by ADMET predictor 10.1 using a modified version of the equation proposed by Austin et al. taking into consideration the compound’s lipophilicity and predicted ionization state at pH 7.4.^47^ This is the default method to predict nonspecific binding to hepatocytes in GastroPlus/ADMET predictor.

To assess the ability of the PBPK model to predict oral absorption and to provide quality control for the IV simulations, predictions were conducted using a “true” unbound intrinsic clearance which was back-calculated from the in vivo clearance. This intrinsic clearance was estimated from the reported in vivo systemic plasma clearance (CL_p_) using the reverse well-stirred model as shown in eqs 3 and 4(48)
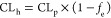
3
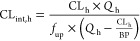
4where CL_h_ is the hepatic clearance, *f*_e_ is the fraction excreted in the urine, *Q*_h_ (in mL/min/kg) is the hepatic blood flow, CL_int,h_ is the unbound hepatic intrinsic clearance (in mL/min/kg), and BP is the blood-to-plasma partitioning ratio. When no information on *f*_e_ was available, *f*_e_ was assumed to be zero. The physiological scaling factors used for this estimation were based on an average weight of rat of 0.25 kg with a liver blood flow of 60 mL/min/kg. When the hepatic blood clearance (CL_h,blood_ = CL_p_/BP) exceeded the liver blood flow, the intrinsic clearance was not calculated and the compounds were excluded from this analysis. For the PBPK simulations, CL_int,h_ was converted into unbound intrinsic clearance in hepatocytes using eq 5 to derive the input parameter, assuming a liver weight (LW) of 40 g/kg and a hepatocytes per gram of liver (HPGL) of 120 10^6^ cells/g liver.
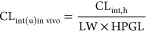
5

It is important to note that this method assumes that the clearance of the selected compound is predominantly due to hepatic metabolism and, to a certain extent, renal clearance. This might not be true for all of the compounds; however, as a method for early discovery, it is believed to be reasonable.

### PBPK Simulations

All PBPK simulations were conducted in GastroPlus 9.8. A previously described whole-body PBPK model for the rat has been developed for generic application and was applied in this study.^49^ The model includes 11 tissue compartments (adipose, bone, brain, gut, heart, kidney, liver, lung, muscle, skin, and spleen). *V*_ss_ was predicted using the modified Rodgers and Rowland method by Lukacova et al.^18,50^ Oral absorption was simulated using the ACAT model, which was combined with the aforementioned full PBPK model for drug disposition. The simulations applied the GastroPlus model for intestinal solubility, which accounts for the enhancement due to bile salt solubilization. The solubilization ratio was estimated within GastroPlus based on the input FaSSIF and FeSSIF solubilities and was then used in the default GastroPlus fasted state ACAT model, which includes values for regional bile salt concentrations in the rat. The immediate-release suspension formulation option was chosen with a particle diameter of 50 μm for all simulations.

For each compound, study and study arm, the single-dose PK in rats was simulated using the respective dosing information and six sets of simulations were conducted for each IV and PO experiment using the different clearance estimation methods: direct scaling, dilution method, unbound, in silico Austin, ML (see below), and back-calculated.

PBPK predictions were also evaluated for an additional set of simulations which used only in silico input parameters predicted with ADMET predictor version 10.1. For clearance, the input parameter was the total Rat Liver Microsomal Clint (CYP_RLM_CL_int_) predicted with an ANNE regression model, built using unbound intrinsic clearance data for model training (*n* = 1431) and testing (*n* = 358). This model, created by Simulations Plus, is based on data collected from various databases and original literature and is reported to have a root-mean-square error (RMSE) of 0.409 μL/min/mg protein. For all of the other input parameters, the GastroPlus default settings were used (i.e., “use predicted” when importing into the GastroPlus database).

Finally, two additional sets of simulations were conducted to evaluate the effect of ML-predicted absorption parameters (solubility, permeability, lipophilicity), without the confounding effect of clearance prediction. These used the back-calculated clearance as input and either in vitro measured or ML-predicted (ADMET predictor v10.1) absorption parameters.

### HT-PBPK Simulations and Comparison with PBPK Simulations

A comparison was also performed between the PO simulations from GastroPlus and the simulations using the high-throughput PK module (HTPK) module in ADMET predictor 10.1 when based on the same input parameters (in vitro measured properties and back-calculated intrinsic clearance). Like GastroPlus, the HTPK model uses the ACAT model for absorption but models disposition with a single central compartment instead of the whole-body PBPK model implemented in GastroPlus. The central compartment volume was set to “mechanistic”, which means that it is equal to the *V*_ss_ estimated using the Rodgers and Rowland method as modified by Lukacova et al.^50^ The advantage of using a reduced model is a significantly reduced computation time compared to the full PBPK model.

### Data Analysis

Data manipulation, analysis, and error metrics calculation were conducted in R version 3.5.1^51^ (using the dplyr, caret, and Modelmetrics packages). Plots were generated using ggplot2, ggpubr, and ggsci packages.

### Criteria for the Evaluation of Prediction Success

The evaluation of prediction success used the metrics recently described by Margolskee and co-workers.^25^ The percentage of predicted parameters within “*x*” fold (e.g., % 2fe, % 3fe, %10fe) of observed gives a useful impression of the overall accuracy and has been widely used in the assessment of PK parameter predictions. The average fold error (AFE) gives an insight into inaccuracy and possible prediction bias, while absolute average fold error (AAFE) gives an insight into prediction precision. Spearman correlation coefficient (ρ) indicates the association between values based on their ranking, which is of great relevance in early discovery settings where the appropriate ranking of compounds is of interest. Root-mean-square loss error (RMSLE) and concordance correlation coefficient (CCC) were also included in the analysis.

## Results

### Data Retrieval, Curation, and Compound Properties

A total of 240 (PO) and 271 (IV) compounds were identified that meet the inclusion criteria with the required PK parameters and all necessary in vitro input data (i.e., aqueous solubility, Log *D*, *P*_eff_, *f*_up_, and CL_heps_). For several of these compounds, more than one study arm was identified (i.e., different dose levels, different experiments), which translated to a total of 432 IV and 480 PO study arms for which separate PBPK simulations were conducted. The datasets with the input parameters and observed PK parameters used for the simulations can be found in the Supporting Information.

The identified compounds represented a diverse set of chemical classes. Using the ring-anchored scaffold system, the structural clustering identified 57 scaffold classes and 27 singletons (clusters that consist of only one compound), while the fingerprint clustering method identified 34 classes with 41 singletons. Further details of the chemical chemotype and subclass composition can be found in Tables S1 and S2 in the Supporting Information.

**Figure 1:**
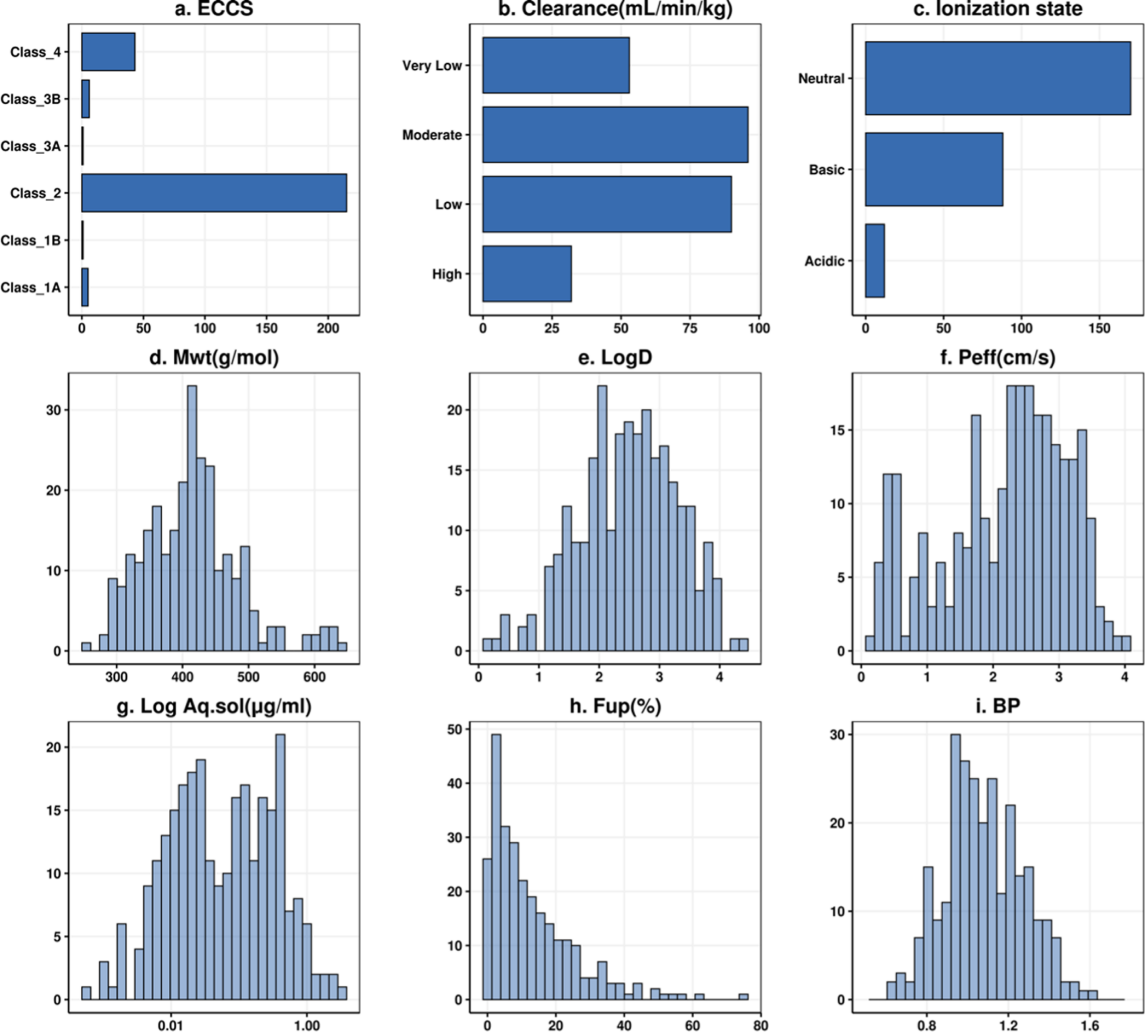
Distribution of compounds in the dataset according to their molecular properties. In (a)–(c), the *y* axis shows the compound classification, while the *x* axis shows the number of compounds. In (d)–(i), the *x* axis shows the value of the molecular property, while the *y* axis shows the number of compounds. *P*_eff_ units are cm/s × 10^–4^.

An overview of the compound properties is shown in Figure 1. The majority of compounds were predicted to belong to class 2 of the ECCS (*n* = 215), suggesting that hepatic metabolism is the main route of elimination. This classification is driven by (i) the ionization state classification (most of the compounds are basic (*n* = 88) or neutral (*n* = 170) at pH 7.4), (ii) the molecular weight distribution (the mean value is 413 Da (>400 Da)), and (iii) the human *P*_eff_ mean value of 2.18 × 10^–4^ cm/s and thus mostly highly permeable compounds. Mean values of the aqueous solubility and Log *D* are 0.20 μg/mL and 2.48, respectively, and most compounds show low to moderate in vivo clearance. The fraction of unbound drug shows a left skewed distribution toward a higher number of compounds with unbound fraction in plasma of <50%. Most compounds (*n* = 236) show moderate binding (*f*_up_ ≥ 2%), while a minority (*n* = 31) show high affinity to plasma proteins (*f*_up_ < 2%).

### PBPK Predictions of IV PK in Rats

**Figure 2:**
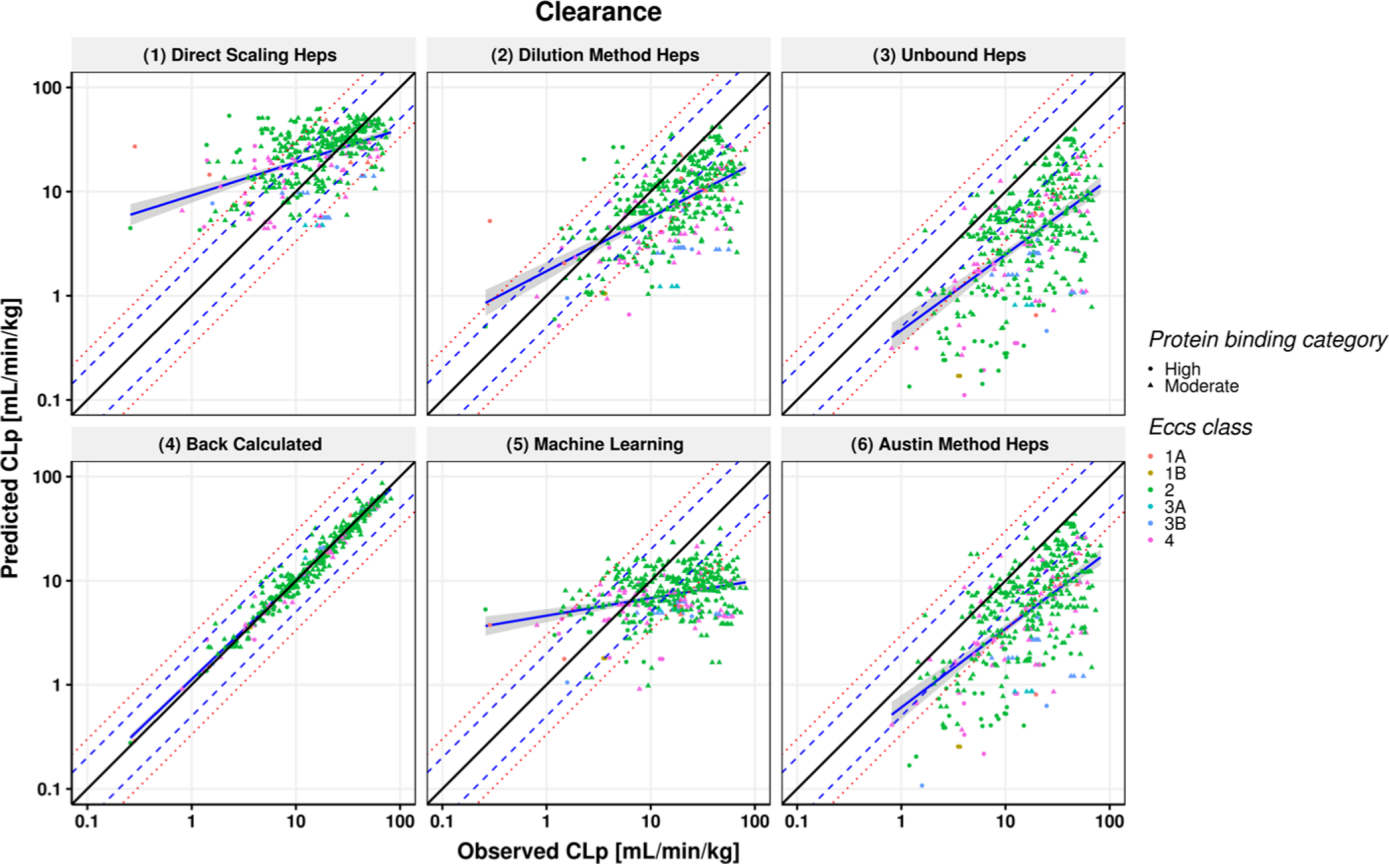
Scatter plots showing the predictions for clearance after IV dosing using six different scaling methods. Observed PK parameters are plotted on the *x* axis while predicted parameters are on the *y* axis. Solid black line represent the line of unity; blue dashed line and red dotted lines represent 2- and 3-fold errors, respectively; blue solid line and shaded gray area represent a linear regression and its 95% confidence interval; and the high and moderate protein binding category compounds are represented by circles and triangles, respectively.

**Figure 3:**
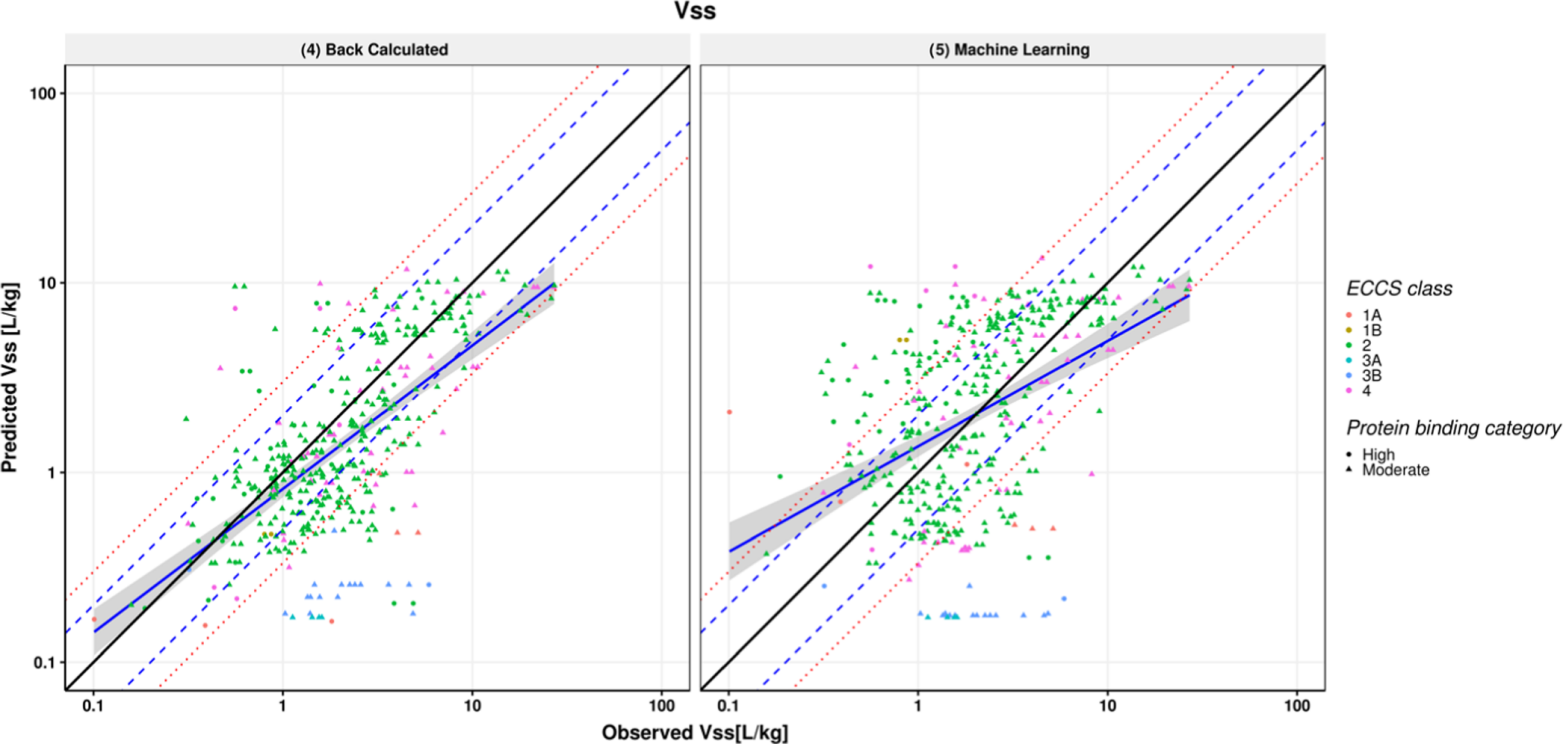
Scatter plot showing predictions for volume of distribution (*V*_ss_) after IV dosing. Observed PK parameters are plotted on the *x* axis, while predicted parameters are on the *y* axis. The solid black line represents the line of unity; blue dashed line and red dotted lines represent 2- and 3-fold error, respectively; blue solid line and shaded gray area represent a linear regression and its 95% confidence interval; and the high and moderate protein binding category compounds are represented by circles and triangles, respectively.

The comparison of predicted and observed PK parameters after IV administration in the rat is presented in Figures 2, 3, and S1. The corresponding error metrics are presented in Table 1. Predictions using the back-calculated clearance are included as a reference for the evaluation of the scaling approach and the physiological parameters used. When predicting clearance and AUC using hepatocytes, the direct scaling and dilution methods both showed relatively good results. In terms of fold error predictions and RMSLE, the direct scaling method showed a slightly better performance in predicting clearance than the dilution method, with a percentage of the predictions within 2-fold errors of 57.6 and 41.7% and RMSLEs of 0.842 and 1.02, respectively (Figure 2 and Table 1). Both methods showed a similar concordance with the observations, the CCC was 0.398 vs 0.423, respectively. Taking the absolute spread of the predictions into consideration, the AAFE values were similar at 2.05 for the direct scaling and 2.53 for dilution methods. In contrast, the bias, represented by the AFE, was 1.42 for direct scaling and 0.463 for dilution methods, which suggests a trend to overprediction of the clearance for the direct scaling methods and to underprediction for the dilution method.

**Table 1:** Error Metrics of the IV Parameters Predictions for the Six Different Simulations

**parameter**	**error metric**	(1) direct scaling	(2) dilution	(3) unbound	(4) back-calculated	(5) machine learning^a^	(6) Austin
CL (mL/min/kg) (*n* = 432)	% 2fe	57.6	41.7	22.5	98.8	35.9	33.3
	% 3fe	76.4	63	38.9	100	60.2	50.9
	AFE	1.42	0.463	0.212	1	0.476	0.302
	AAFE	2.05	2.53	4.81	1.13	2.76	3.48
	RMSLE	0.842	1.02	1.46	0.165	1.1	1.24
	CCC(log)	0.398	0.423	0.309	0.981	0.176	0.397
	ρ	0.471	0.541	0.528	0.98	0.246	0.574
	*R*2	0.179	0.198	0.181	0.952	0.0391	0.217
	*R*2(log)	0.222	0.33	0.379	0.964	0.0902	0.419
AUC_inf_ (ng· h/mL) (*n* = 432)	%2fe	57.6	41.4	22.9	98.8	36.1	33.3
	%3fe	76.4	63	38.9	100	60.2	50.9
	AFE	0.703	2.16	4.71	1	2.1	3.31
	AAFE	2.05	2.53	4.81	1.14	2.76	3.48
	RMSLE	0.949	1.15	1.86	0.187	1.22	1.53
	CCC(Log)	0.603	0.545	0.364	0.986	0.422	0.464
	ρ	0.6222	0.638	0.564	0.982	0.489	0.611
	*R*2	0.0782	0.216	0.401	0.974	0.129	0.353
	*R*2(log)	0.419	0.471	0.436	0.972	0.308	0.489
*V*_ss_ (L/kg) (*n* = 423)	% 2fe	59.1	60	60.8	59.8	45.4	60.5
	% 3fe	81.6	82	82.3	81.3	70.4	82
	AFE	0.692	0.702	0.704	0.694	1.01	0.703
	AAFE	2.01	2	2	2.02	2.45	2
	RMSLE	0.538	0.538	0.539	0.542	0.663	0.539
	CCC(Log)	0.582	0.584	0.584	0.576	0.412	0.584
	ρ	0.603	0.602	0.602	0.598	0.46	0.602
	*R*2	0.449	0.447	0.446	0.425	0.29	0.447
	*R*2(log)	0.401	0.4	0.399	0.392	0.182	0.399

a Machine learning column also uses ML for *f*_up_ and Log *D* not just for clearance.

The extent of protein binding was an indicator of prediction success for the different CL methods, as summarized in Table 2, where the highly protein-bound compounds are less accurately predicted using the direct scaling (AAFE = 4.21) compared to the moderately bound compounds (AAFE = 1.86). For the dilution method, on the other hand, compounds were similarly predicted irrespective of their protein binding category with AAFEs of 2.53 and 2.54 for both classes, additional error metrics can be found in Table S3. Prediction success also varied with the clearance class, as shown in Table S4. Predictions within 2-fold error for the direct scaling method were 82.5 and 67.3% for moderate to high clearance compounds respectively, compared to 48.3 and 18.8% for the low and very low clearance categories. In contrast, the dilution method performs better in the low to very low clearance range, with 53.1 and 56.2% of the predictions within 2-fold, although the prediction success in the moderate to high clearance range is lower than for direct scaling. All of the IV error metrics calculated for the six scaling methods classified according to protein binding and clearance category are presented in the Supporting Information (Tables S3 and S4). For the other explored scaling methods, the prediction success was lower compared with both direct scaling and dilution methods. When assuming that the measured CL_int,heps_ is unbound (fu_inc_ = 1), the prediction accuracy was very low (RMSLE = 1.46) and only 22.5% of the simulations were predicted within 2-fold error, with a general underprediction for the clearance (AFE = 0.212). This was similar as when the in silico Austin was used with an AAFE of 3.48 (Figure 2 and Table 1). CL predictions using the ML CL_int_ as an input (CYP_RLM in ADMET predictor), which were based solely on the compounds’ structure, showed a moderate prediction success, where 35.9 and 60.2% were predicted within 2- and 3-fold errors, respectively. However, the correlation in terms of the spearman correlation coefficient was lower than for all of the other methods (Figure 2 and Table 1).

**Table 2:** Error Metrics of the IV Parameter Prediction by Protein Binding Category

		(1) direct scaling		(2) dilution	
		protein binding category (high: *n* = 50, moderate: *n* = 382)			
parameter	error metric	high	moderate	high	moderate
CL (mL/min/kg)	% 3fe	42%	80.9%	60%	60.3%
	AAFE	4.21	1.86	2.53	2.54
	AFE	0.25	0.805	1.31	2.31
	*R*2	0.137	0.223	0.0536	0.193

*V*_ss_ predictions using the modified Rodgers and Rowland method by Lukacova et al.,^50^ based on a combination of in vitro inputs (*f*_up_, Log *D*) and in silico predicted BP and p*K*_a_s, are shown in Figure 3 and Table 1. The predictions show relatively good agreement with the observations with 59.1% of the predictions within 2-fold and AFE and AAFE of 0.692 and 2.01, respectively. Although the input parameters were the same for all of the scaling methods, with the exception of the unbound CL_int,hep_, small differences in the prediction success were observed for *V*_ss_ across the methods (Table 1). This was expected due to the impact that the extraction ratio from eliminating organs (e.g., liver) has in the prediction of *V*_ss_ using the mechanistic models. Notably, the *V*_ss_ estimations using ML-predicted *f*_up_ and Log *D* showed less success than those based on measured data for these inputs, with the percentage of the predictions falling within 2- and 3-fold errors of 45.4 and 70.4%, respectively, and the AFE and AAFE were 1.01 and 2.45, respectively.

### PBPK Predictions of PO PK in Rats

**Figure 4:**
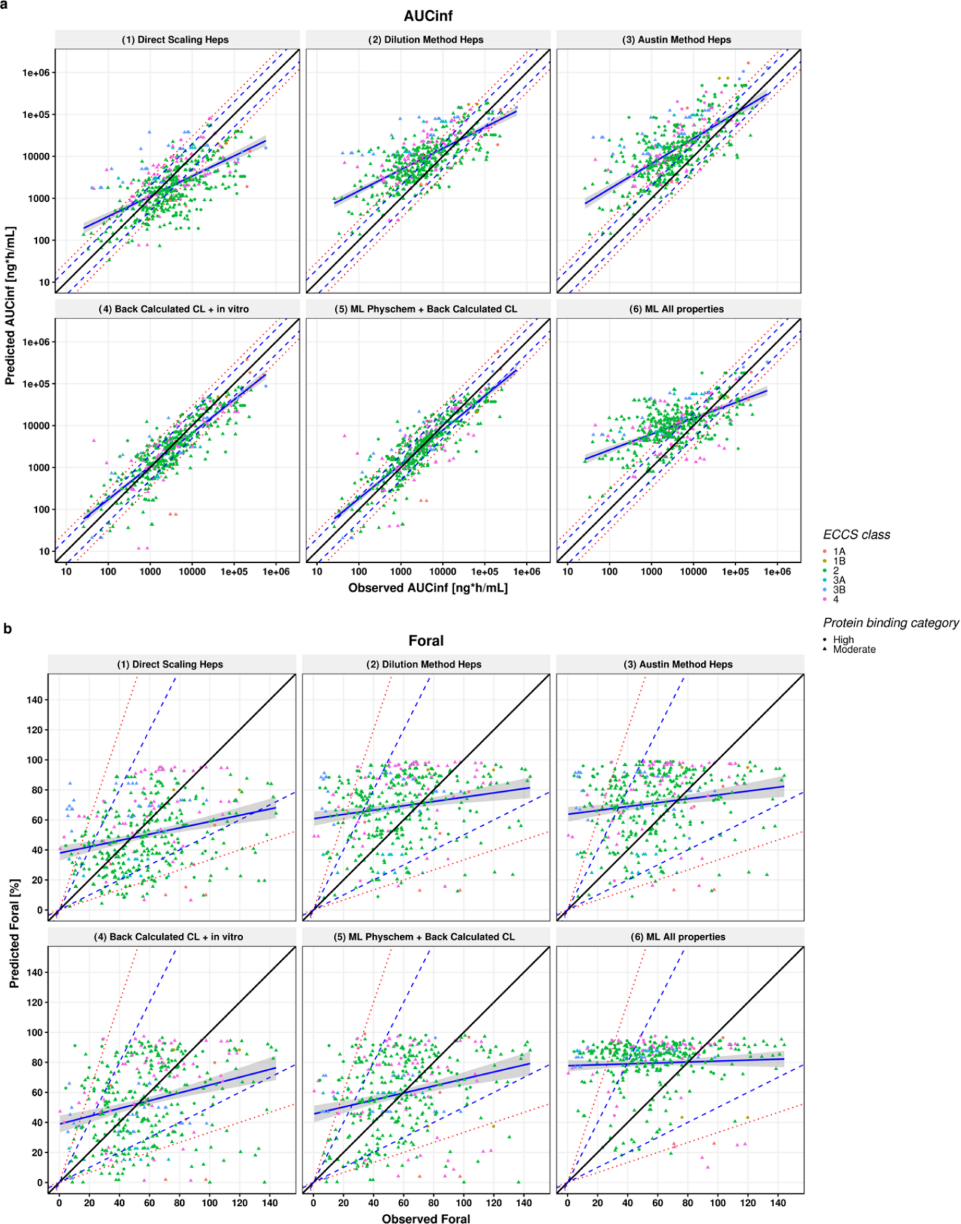
Scatter plots showing (a) AUC_inf_ and (b) *F*_oral_ predictions using five different scaling methods. Observed PK parameters are plotted on the *x* axis, while predicted parameters are on the *y* axis. The solid black line represents the line of unity; blue dashed line and red dotted lines represent 2- and 3-fold errors, respectively; blue solid line and shaded gray area represent a linear regression and its 95% confidence interval; and the high and moderate protein binding category compounds are represented by circles and triangles, respectively.

A comparison between observed and bottom-up PBPK predictions of the PK parameters AUC_inf_, *F*_oral_, and *C*_max_ after PO administration in rats can be found in Figures 4 and S4, whereas the error metrics are summarized in Table 3. When using CL_int,heps_ as input, only results for clearance scaling using the dilution method and direct scaling method are presented here due to the comparatively poor predictions of IV clearance using the other scaling methods. Considering the PO simulations using the aforementioned CL scaling methods, there was a good and similar correlation between observed and predicted AUC_inf_ for both methods (ρ *>* 0.6). AUC_inf_ predictions within 2- and 3-fold errors were also similar at 38 and 56.8% for direct scaling and 31.9% and 50.4% for the dilution method. While the direct scaling method tended toward underprediction of the AUC_inf_ (AFE 0.589), the dilution method tended to overpredict (AFE = 2.62). Nevertheless, both methods showed an acceptable precision of AUC_inf_ predictions (AAFE 3.29 and 3.57 for the direct and dilution scaling methods, respectively). The prediction success of *C*_max_ was in line with the AUC_inf_ predictions. Simulations were within 2- and 3-fold errors for 40.5 and 58% for direct scaling and were within 38.8 and 59% for the dilution method. The bias and precision were different for both methods, where a general trend to overprediction of *C*_max_ was observed for the dilution method. In contrast, the AAFE was similar for both methods and close to 3-fold.

**Table 3:** Error Metrics of the Oral Parameter Prediction for the Six Different Simulations

**parameter**	**error metric**	(1) direct scaling (*n* = 479)	(2) dilution (*n* = 480)	(3) Austin (*n* = 480)	(4) back-calculated CL + in vitro physchem (*n* = 480)	(5) ML physchem + back-calculated CL (*n* = 480)	(6) ML (all properties) (*n* = 480)
AUC_inf_ (ng̣ ·h/mL)	% 2fe	38	31.9	23.3	59.4	63.5	27.9
	% 3fe	56.8	50.4	40.8	80	81.9	45.4
	AFE	0.589	2.62	4.13	0.79	0.905	2.9
	AAFE	3.29	3.57	4.8	2.12	2.01	4.2
	RMSLE	1.53	1.6	1.93	1.1	1.03	1.8
	CCC(Log)	0.559	0.55	0.502	0.801	0.825	0.417
	ρ	0.6	0.673	0.662	0.855	0.858	0.512
	*R*2	0.075	0.254	0.229	0.384	0.497	0.475
	*R*2(log)	0.367	0.473	0.477	0.654	0.682	0.322
*C*_max_ (ng/mL)	% 2fe	40.5	38.8	36.9	47.5	48.1	33.5
	% 3fe	58	59	54.6	72.5	66.2	50.4
	AFE	0.884	2.13	2.51	1.03	1.53	2.41
	AAFE	2.97	3.12	3.34	2.46	2.53	3.69
	RMSLE	1.36	1.45	1.54	1.16	1.21	1.65
	CCC(Log)	0.563	0.549	0.532	0.713	0.715	0.453
	ρ	0.561	0.618	0.622	0.755	0.758	0.531
	*R*2	0.111	0.206	0.273	0.359	0.447	0.133
	*R*2(log)	0.32	0.395	0.408	0.514	0.555	0.289
*F*_oral_	% 2fe	66.3	68.6	68.6	64.5	68.1	65.9
	% 3fe	84.9	85.4	85.2	83	84.7	82.7
	AFE	0.83	1.22	1.26	0.808	0.928	1.46
	AAFE	1.89	1.85	1.88	2.05	1.95	1.94
	RMSLE	0.844	0.824	0.836	0.959	0.909	0.873
	CCC(lin)	0.0607	0.0515	0.0491	0.0724	0.0743	0.0205
	ρ	0.307	0.257	0.221	0.309	0.307	0.157
	*R*2	0.0227	0.0161	0.0142	0.0241	0.0253	0.00425
	*R*2(log)	0.0477	0.0218	0.018	0.0547	0.053	0.0016

*F*_oral_ predictions were within the 2- and 3-fold range for 66.3–84.9% of the simulations using direct scaling and for 68.6 and 85.4% when using the dilution method. The bias for *F*_oral_ was within 0.83- to 1.22-fold for direct and dilution methods, and the overall AAFE was less than 2-fold (Table 3). Nevertheless, the prediction correlation was poor both in terms of *R*2, spearman, and CCC (Table 3). Considering the spread of the measured *F*_oral_ data and the limited range for this parameter (from 0 to 140%), the prediction success in terms of overall bias and precision and lack of correlation was expected. All of the other scaling methods had similar performance in terms of *F*_oral_ predictions.

To assess the prediction success for oral PK parameters without the confounding factor of hepatic clearance prediction, PO predictions were made using a CL_int(u),in vivo_ back-calculated from the observed systemic CL. When the in vitro measured physicochemical properties were used, namely, Log *D*, aqueous solubility, permeability, and FaSSIF and FeSSIF solubility (when available), a high degree of agreement between predicted and observed AUC_inf_ and *C*_max_ was seen as shown in Figures 4 and S2 and Table 3. When accounting for the “right” clearance, the percentage of predictions within 2- and 3-fold error increased to 59.4 and 80% for AUC_inf_ and to 47.5 and 72.5% for *C*_max_. In terms of overall bias, AUC_inf_ and *C*_max_ were generally predicted within 2-fold (AFE of 0.79 and 1.03 for AUC_inf_ and *C*_max_, respectively) and the correlations between the measured and predicted AUC_inf_ and *C*_max_ values were strong with CCC values of 0.801 and 0.713 and ρ of 0.85 and 0.75. This suggests that the bottom-up PBPK approach allows good predictions of the PK when the clearance can be well predicted. The success of *F*_oral_ did not improve compared to the fully bottom-up predictions.

**Figure 5:**
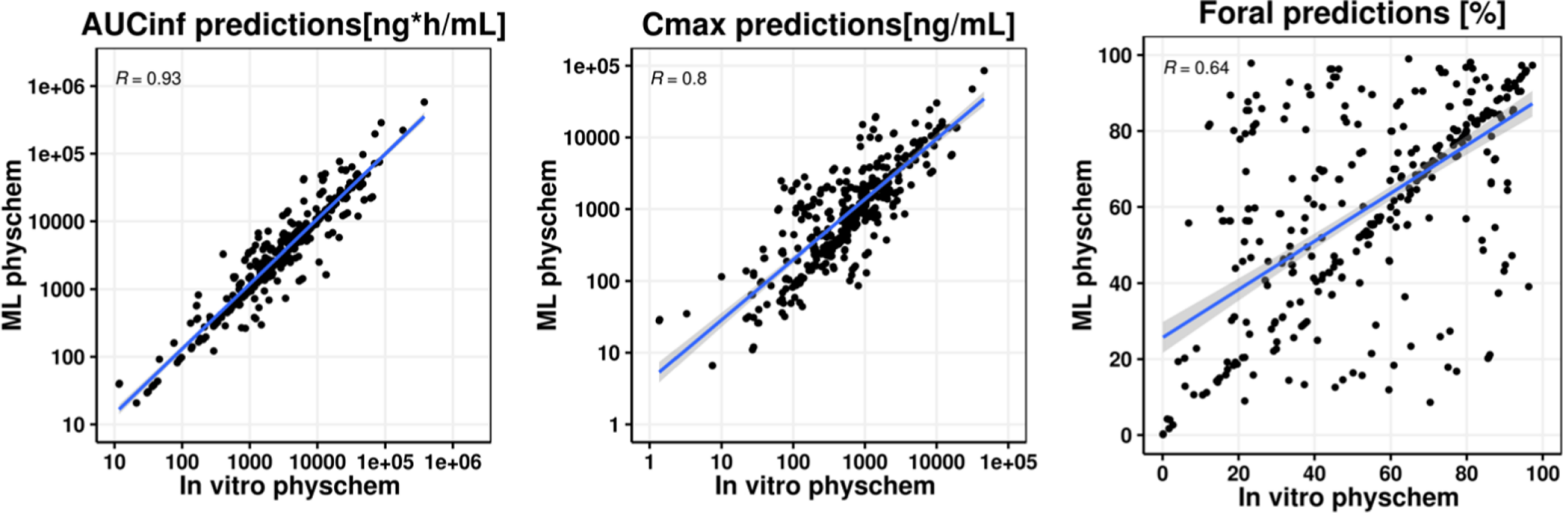
Scatter plots comparing AUC_inf_, *C*_max_, and *F*_oral_ predictions of the back-calculated clearance scaling method using the in vitro physicochemical properties (*x*-axis) vs the machine learning predicted properties (*y*-axis). Blue solid line and shaded gray area represent the linear regression and its 95% confidence interval.

Repeating the simulations using a back-calculated clearance but with ML-predicted physicochemical properties as inputs for oral absorption showed that the predictions within 2- and 3-fold errors for AUC_inf_ were 63.5 and 81.9% compared to 59.4% and 80% for measured inputs. The AAFE for AUC_inf_ was overall reduced to 2.01 compared to 2.12 for measured inputs. In addition, the correlation and the concordance coefficients were strong when using the ML inputs (CCC = 0.825 and 0.715, ρ = 0.858 and 0.758) for AUC_inf_ and *C*_max_, respectively. Given the minimal differences between the error metrics for these two simulations, a head-to-head comparison was conducted. As can be seen in Figure 5, there are minimal differences in predictions except for *F*_oral_ predictions. Further examination comparing the ML-predicted properties in ADMET predictor 10.1 vs the measured parameters (aqueous solubility, Log *D*, *P*_eff_, and *f*_up_) revealed a good correlation between observed and predicted Log *D*, *P*_eff_, and *f*_up_, whereas a poor correlation was observed for solubility (Figure S3).

### Comparison between PBPK Simulations and HT-PBPK Simulations

**Figure 6:**
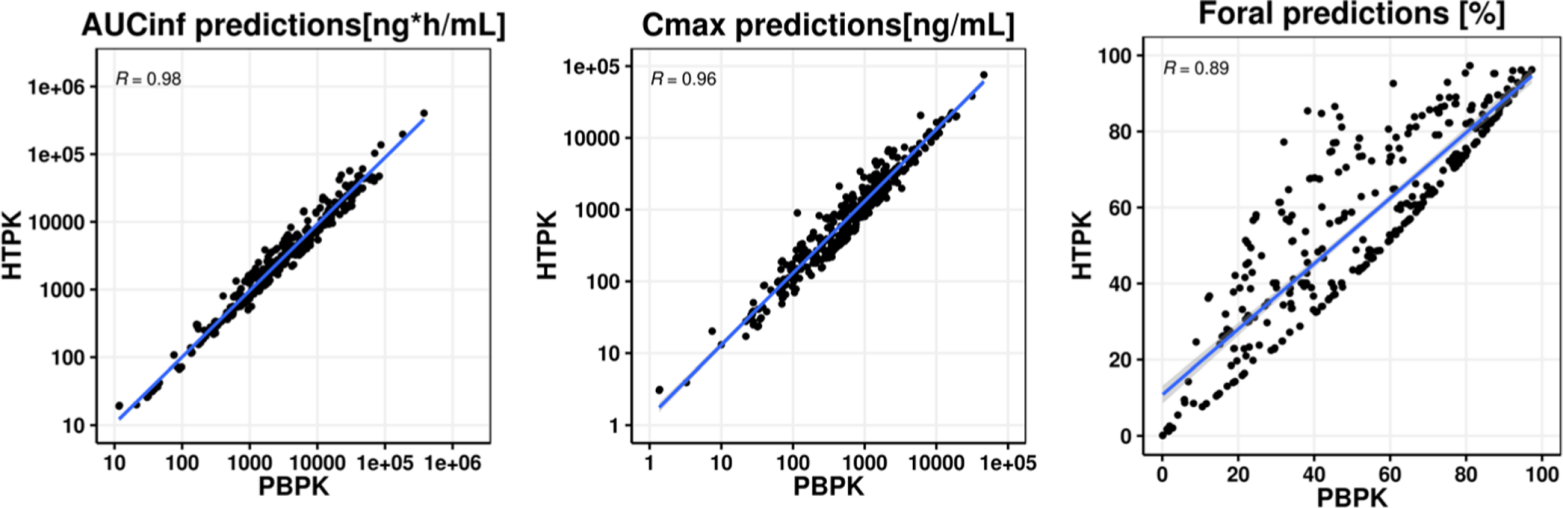
Scatter plots comparing AUC_inf_, *C*_max_, and *F*_oral_ predictions of the back-calculated clearance scaling method using the PBPK module (*x*-axis) vs the HTPK module (*y*-axis). Blue solid line and shaded gray area represent the linear regression and its 95% confidence interval, respectively.

A comparison was made between predictions using the full PBPK model and ACAT model in GastroPlus 9.8 and the HTPK module in ADMET predictor 10.1. The same set of input parameters was used for both, namely, the in vitro measured properties and the back-calculated intrinsic clearance. As may be seen in Figure 6, predictions of AUC, *C*_max_, and *F*_oral_ using the HTPK module were similar to the predictions using GastroPlus although minor differences could be observed, especially with regard to *F*_oral_. Both GastroPlus and HTPK simulations were run on a machine with an Intel R processor running at 2.40 GHz using 16 MB of RAM, but despite this, there was a big difference in calculation time. Using the GastroPlus software and the full PBPK and ACAT models, the total runtime was approximately 3.5 h for PO (and IV) simulations, including the time it took to import the structures and create the database. In contrast, using the HTPK module in ADMET predictor 10.1, the same process took approximately 10 s.

## Discussion

Only a few studies have focused on the evaluation of bottom-up PBPK approaches in preclinical stages and their application in early drug discovery.^33,34^ Parrott et al. evaluated the utility of such approaches to predict PK plasma profiles in rat for 68 compounds, while Daga et al. explored several clearance scaling approaches for the prediction of bioavailability in rat. In this work, we present a comprehensive analysis on the evaluation of bottom-up PBPK approaches for the prediction of rat PK parameters in an early discovery setting. The work is demonstrated on a considerably larger library of diverse compounds for both IV and oral routes (270 and 240 compounds, respectively).

One of the advantages of the dataset presented in this work is the availability of all of the in vivo PK parameters and the most significant in vitro physicochemical properties for all of the compounds. Compiling such a dataset comprising a significant number of diverse compounds with these available measurements is a necessary step toward improving PBPK models. This allowed for the implementation of key importance in vitro measurements in the models such as the fraction of drug unbound in the rat plasma and primary hepatocytes intrinsic clearance, compared to other commonly used measurements such as the microsomal clearance, which might not provide the required sensitivity for low clearance compounds.

Other studies have compiled similar datasets; however, most of these studies were performed on a large scale, using cross-company combined datasets and thus including experimental measurements from different sources.^24,25^ While the interlab differences and discrepancies within in vitro assays and the lack of class-specific corrections might be the limitations of such batch approaches, they offer a larger coverage of the compounds’ chemical space and provide more confidence in PBPK models within the discovery project teams.

Our analysis has also shown that correct estimation of clearance is a key factor affecting prediction accuracy, emphasizing the impact of the clearance scaling approaches and other physiological/physicochemical input parameters. For example, the assumption that the measured drug CL_int,hep_ in vitro is unbound showed poor performance in the prediction of both IV and PO parameters, a common approach in early drug discovery. The direct scaling and dilution methods showed similar overall performance; however, the direct scaling seemed to work better for less tightly protein-bound compounds (AAFE = 1.86 for moderate binding vs 4.21 for high binding). Uncovering such differences in prediction accuracy with scaling approach is important to build an understanding of the influence of physicochemical and metabolic properties on optimally predictive PBPK modeling of project compounds. Analysis of trends for larger collections of compounds can lead to guidance and best practices on how to implement the most appropriate scaling method in early-discovery PBPK modeling.

A back-calculated hepatic clearance, along with in vitro measured properties was used to evaluate the model’s ability to predict oral PK parameters without the confounding effect of inaccuracy in clearance and hepatic first-pass predictions. This approach achieved the highest prediction accuracy, with an AAFE < 2.5 and AFE < 1.5 for all oral parameters assessed (AUC_inf_, *C*_max_, and *F*_oral_). The performance of the PBPK models incorporating the back-calculated clearance when in vitro measured inputs were replaced by in silico predicted properties was also evaluated. Interestingly, despite poor predictions of some of the molecular properties, particularly solubility-related inputs such as aqueous solubility (Figure S3), very good concordance was seen between these two sets of predictions (Figure 5 and Table 3). This might be attributed to an overall low sensitivity of the simulations for compounds in our dataset to solubility, the relatively high permeability, and the relatively accurate prediction of parameter such as Log *D* and *P*_eff_ using ADMET predictor 10.1 (Figure S3). Further scrutiny of the simulations indicates a bias toward the prediction of a high fraction absorbed (*F*_abs_), independent of whether measured or ML inputs are used (Figure S6). When using measured inputs 267 out of 480 data points had a simulated *F*_abs_ > 90% and the mean *F*_abs_ was 79%, whereas when using ML inputs 368 out of 480 have *F*_abs_ > 90% and the mean *F*_abs_ was to 89%. Given that simulated *F*_abs_ values were high for the majority of data points the overall sensitivity to the input parameter defining oral absorption in the PBPK model was low. This might explain the limited differences between using ML and measured input parameters and the significant improvement of the predictions when using the back-calculated clearance as an input for the simulation.

Additional challenges limiting wider use of in silico PBPK tools could be the difficulty, the lack of expertise in the use of the models, and the time consumption factor. Therefore, successful predictions obtained from HT-PBPK models such the HTPK module in ADMET predictor could provide rapid PBPK assessment (7.82 s for 480 oral study arms) and optimize modeling time. Overall, the implementation of HT-PBPK in drug discovery can provide a balance between effectiveness and efficiency of the PBPK modeling process.

The work presented herein is focused on rat predictions, as such predictions might be limited for the direct prediction of human pharmacokinetics, yet for the purpose of compound prioritization in early drug discovery, nonclinical species PK remain valuable as a means of focusing the discovery efforts on the most promising candidates and to assess further developability when progressing compounds to repeat dose toxicological and safety pharmacology studies, which are a prerequisite to enable phase 1 studies. Furthermore, the learnings obtained in this work with respect to the IVIVE strategy, scaling approaches, and the use of the right in vitro systems can be extrapolated to the human PBPK predictions.^12,14^

Finally, integration of ML approaches for clearance predictions in the LI/LO phases could vastly accelerate the drug discovery process through optimization of the compound’s chemical structure prior to synthesis. However, further effort is required to improve the prediction success using these models. While several general models have been recently described in the literature, the development of a local model might have better applicability for the approach described herein, and this is an area for further development.

## Conclusions

An evaluation of bottom-up PBPK predictions in the rat including a comparative analysis of clearance scaling approaches has been performed. Accuracy of clearance prediction was critical and the optimal clearance scaling approach for a compound was influenced by its molecular properties. In particular, careful consideration of the plasma protein binding could improve the accuracy of model predictions. The use of a back-calculated hepatic clearance showed that, if a good estimate of clearance is achieved, then bottom-up PBPK predictions from minimal measured in vitro data can be useful for compound ranking. The use of ML approach was successful when used for the physicochemical properties but not for the clearance, where the ML all properties method did not show the accuracy required. Improvement of this approach can be established through expanding the training sets behind the PBPK clearance models and will be considered for future implementation. The establishment of HT-PBPK modeling approaches in drug discovery can accelerate and facilitate the PBPK modeling procedure and promote its application within the drug discovery process.

## Supporting Information Available

Scatter plots of different predicted vs observed PK parameters and measured vs ML-predicted properties and/or parameters (Figures S1–S5); histogram of predicted *F*_abs_ (Figure S6); scaffold analysis (Table S1); and error metrics by protein binding, clearance category, and complete error metrics for IV and PO simulations (Tables S2–S6) (PDF)
Datasets of IV input parameters for simulations including observed PK parameters (XLSX)
Datasets of PO input parameters for simulations including observed PK parameters (XLSX)
