# Supplementary material for: Evaluation of the Success of High-Throughput Physiologically Based Pharmacokinetic (HT-PBPK) Modeling Predictions to Inform Early Drug Discovery
Source: Mol Pharm. 2022 Apr 27;19(7):2203–16. doi: 10.1021/acs.molpharmaceut.2c00040 (PMC9257750; doi:10.1021/acs.molpharmaceut.2c00040)
Supplement: Supplementary file 1 — mp2c00040_si_001.pdf [file mp2c00040_si_001.pdf]

Supplementary information for:

Evaluation of the success of high throughput physiologically-based pharmacokinetic (HT-PBPK) modeling predictions to inform early drug discovery

Doha Naga[1, 2,$], Neil Parrott[1], Gerhard F. Ecker[2] and Andrés Olivares-Morales[1,$,*]

[1]Roche Pharma Research and Early Development (pRED), Roche Innovation Center Basel, 4070 Basel, Switzerland.
[2]Department of Pharmaceutical Sciences, University of Vienna, 1090 Vienna, Austria.

[$]Both authors contributed equally to this work
*To whom correspondence should be addressed

***Corresponding Author**
Dr. Andrés Olivares-Morales
Roche Pharma Research and Early Development (pRED), Grenzacherstrasse 124, 4070 Basel Switzerland
Phone +41 61 688 0798
Email: andres.olivares@roche.com

Figure S1. Scatter plots showing AUC_{inf} predictions for the six different scaling methods (IV route)

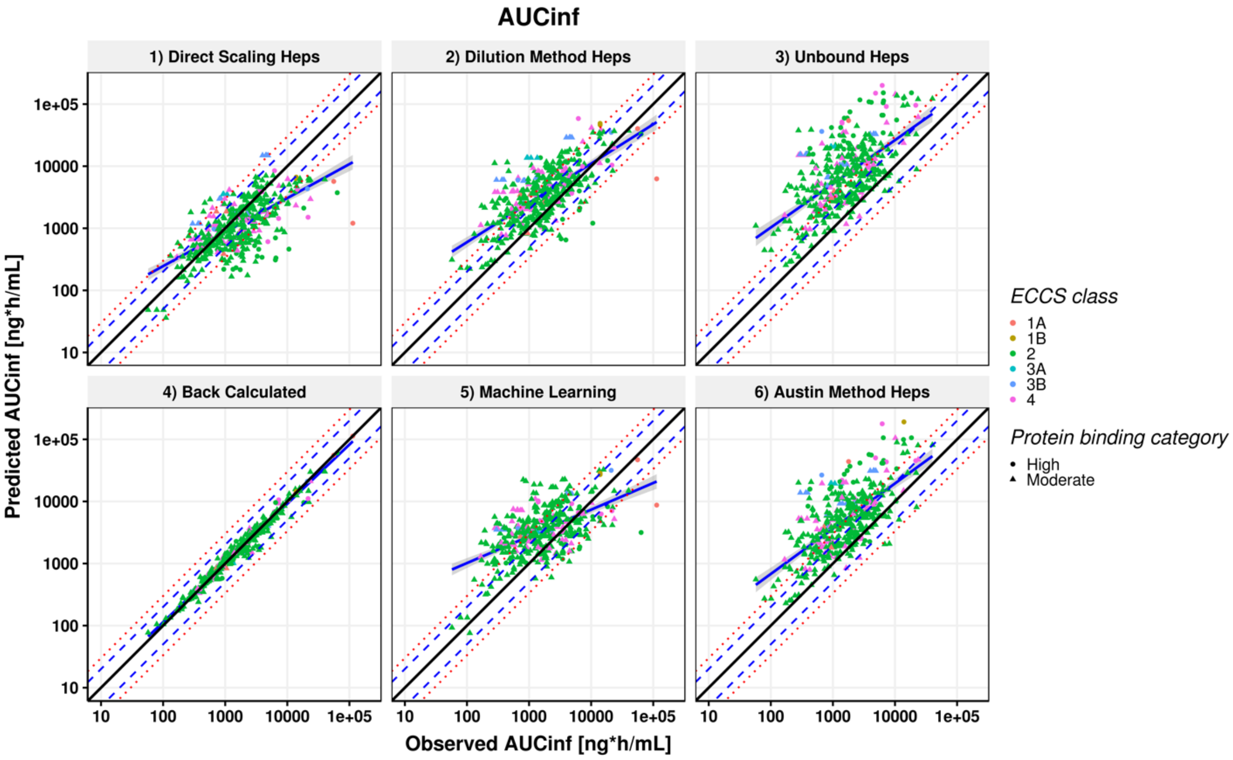

Observed PK parameters are plotted on the x axis while predicted parameters are on the y axis. Solid black line represent the line of unity; blue dashed-line and red dotted lines represents the 2 and 3 fold error, respectively; blue solid line and shaded grey area represents a linear regression and its 95% confidence interval. The high and moderate protein binding category compounds are represented by circles and triangles respectively.

Figure S2. Scatter plots showing C_{max} predictions for the six different scaling methods (PO)

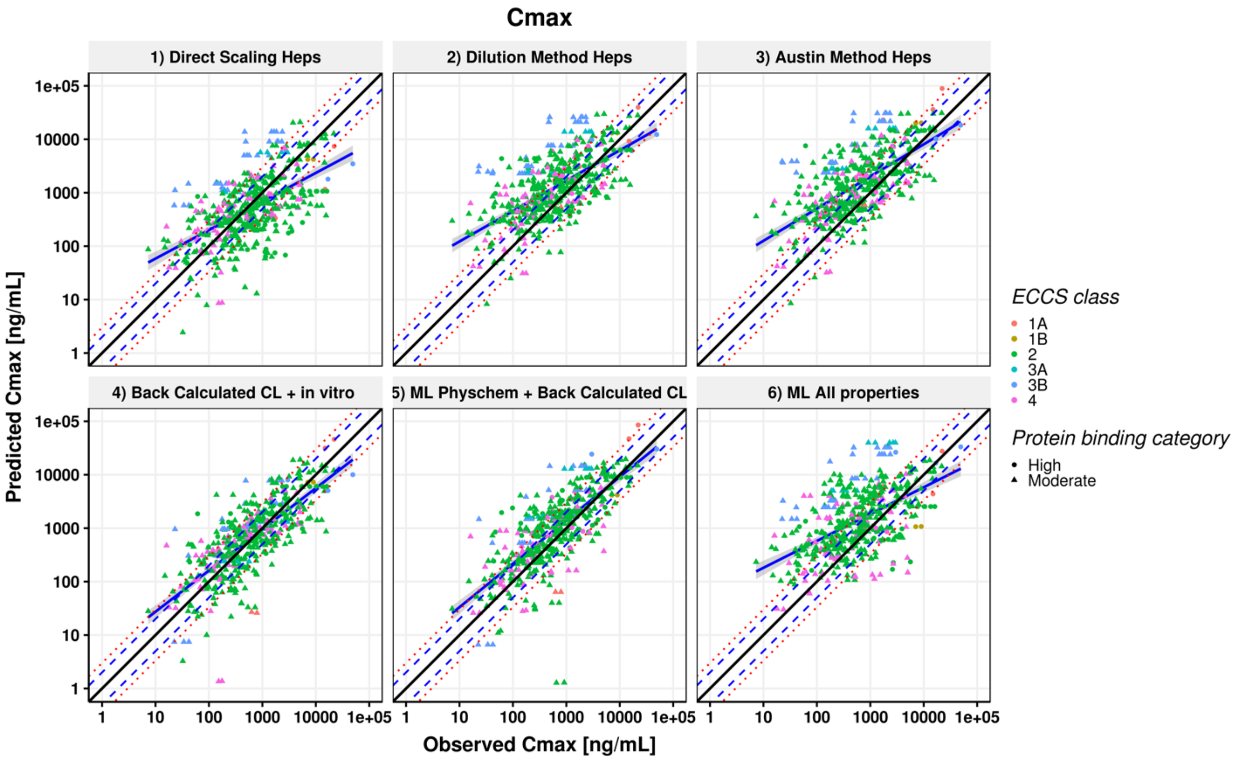

Observed PK parameters are plotted on the x axis while predicted parameters are on the y axis. Solid black line represent the line of unity; blue dashed-line and red dotted lines represents the 2 and 3 fold error, respectively; blue solid line and shaded grey area represents a linear regression and its 95% confidence interval. The high and moderate protein binding category compounds are represented by circles and triangles respectively.

Figure S3. A comparison between the measured (x-axis) and predicted values (y-axis) of the physico chemical properties for the compounds.

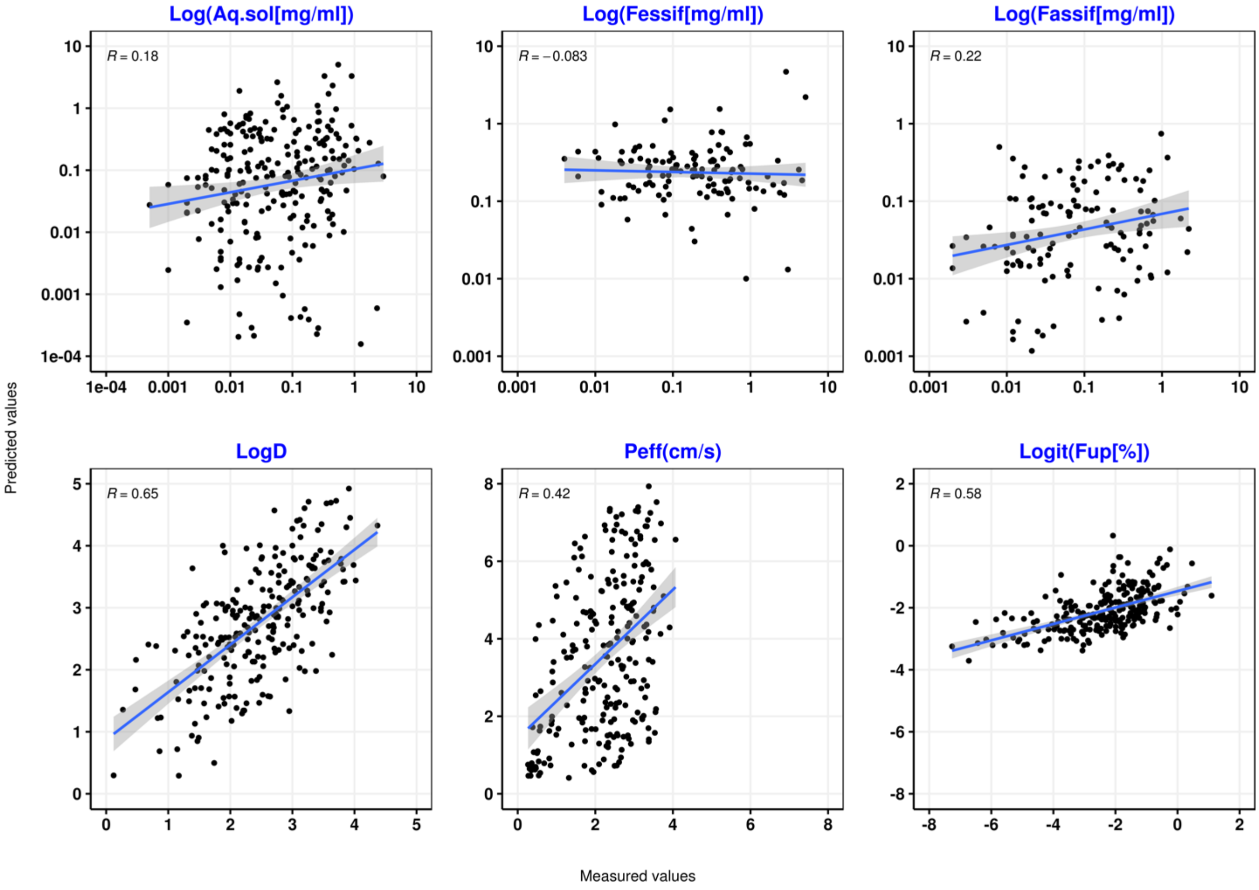

Spearman correlation coefficients are indicated on each plot. Blue lines represent the regression lines. Logit transformation* is applied to the Fup(%). Logit transformation was applied according to the equation : $logit(x) = \frac{x}{100}, \log(\frac{x}{1-x})$

Figure S4 Scatter plot illustrating dose normalized $AUC_{0\text{-inf}}$ predictions for oral formulations.

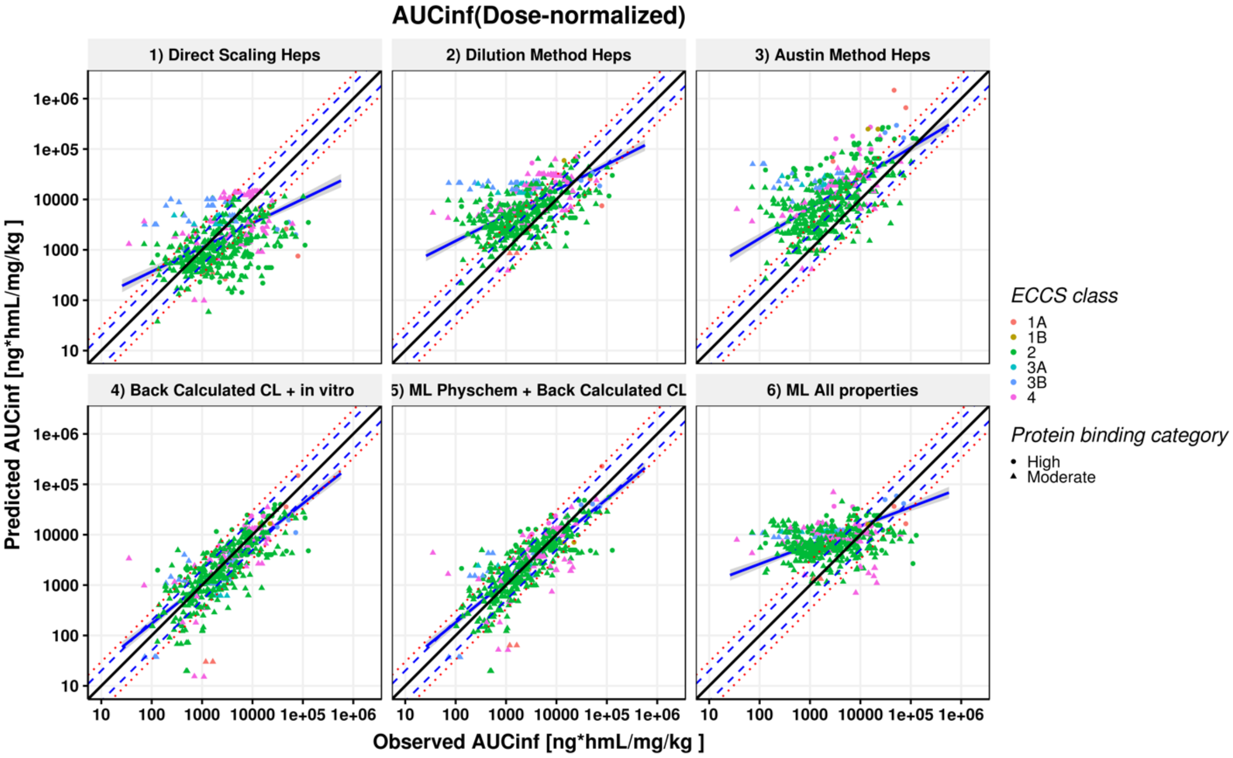

Observed PK parameters are plotted on the x axis while predicted parameters are on the y axis. Solid black line represent the line of unity; blue dashed-line and red dotted lines represents the 2 and 3 fold error, respectively; blue solid line and shaded grey area represents a linear regression and its 95% confidence interval. The high and moderate protein binding category compounds are represented by circles and triangles respectively.

Figure S5. Scatter plot illustrating dose normalized C_{max} predictions for oral formulations.

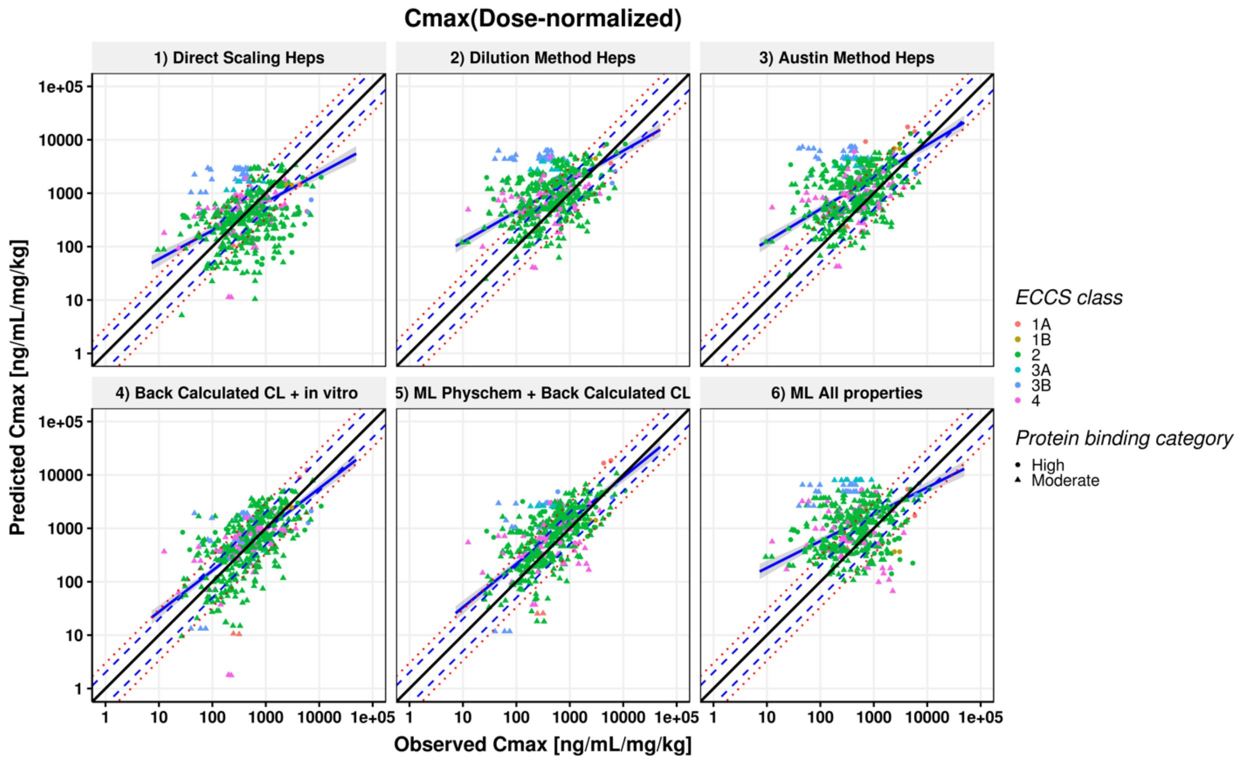

Observed PK parameters are plotted on the x axis while predicted parameters are on the y axis. Solid black line represent the line of unity; blue dashed-line and red dotted lines represents the 2 and 3 fold error, respectively; blue solid line and shaded grey area represents a linear regression and its 95% confidence interval. The high and moderate protein binding category compounds are represented by circles and triangles respectively.

Figure S6. Histogram of predicted fraction absorbed (F_{abs}) in rats after PO administration using back-calculated C_{Lint} and measured vs machine learning predicted input parameters (aqueous solubility, intestinal permeability, LogD and f_{up})

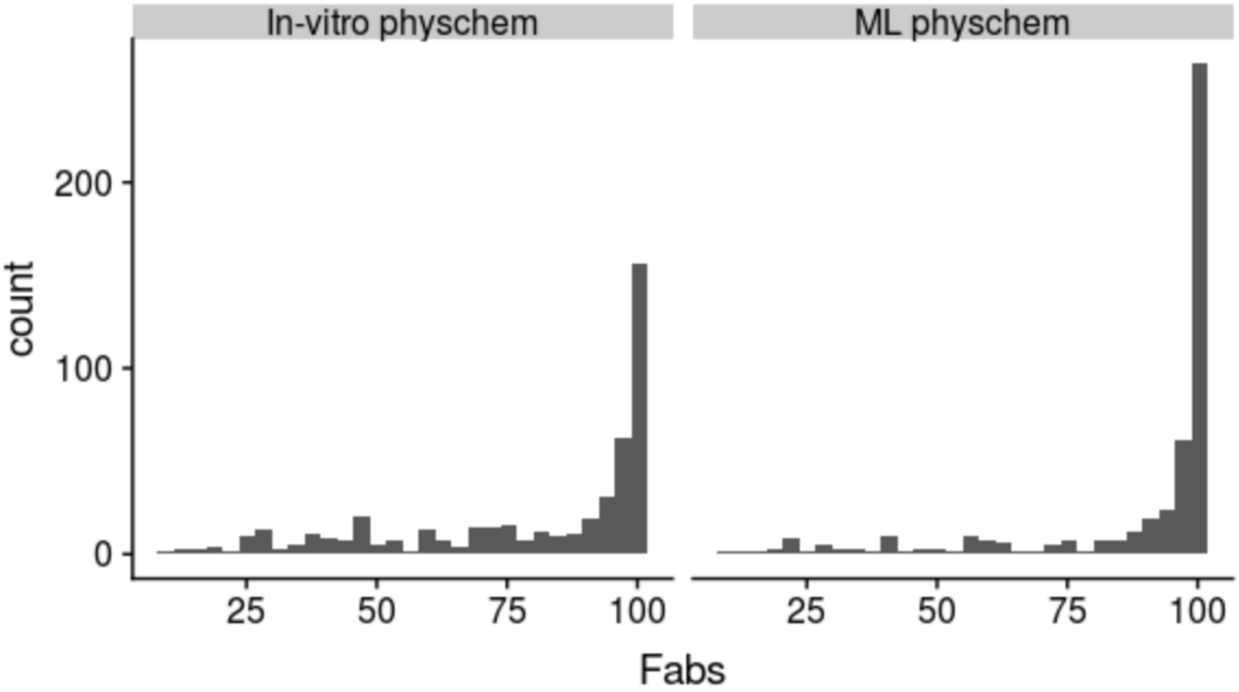

Supplementary tables

Table S1. Ring scaffold system classes.

**Class ID**	**Class Size**
Class identifier. ID values of non-singleton classes generally have the form "C 1", "C 2", etc., while singleton classes have "S 1", "S 2", etc.	Number of compounds in the class
C 1	23
C 2	21
C 3	19
C 4	16
C 5	14
C 6	14
C 7	14
C 8	11
C 9	10
C 10	9
C 11	8
C 12	7
C 13	7
C 14	7
C 15	6
C 16	6
C 17	6
C 18	6
C 19	5
C 20	5
C 21	5
C 22	5
C 23	5
C 24	4
C 25	4
C 26	4
C 27	4
C 28	4
C 29	4
C 30	4
C 31	4
C 32	4
C 33	4
C 34	4
C 35	4
C 36	3
C 37	3
C 38	3
C 39	3
C 40	3
C 41	3

C 42	3
C 43	3
C 44	3
C 45	3
C 46	3
C 47	2
C 48	2
C 49	2
C 50	2
C 51	2
C 52	2
C 53	2
C 54	2
C 55	2
C 56	2
C 57	2
S 1	1
S 2	1
S 3	1
S 4	1
S 5	1
S 6	1
S 7	1
S 8	1
S 9	1
S 10	1
S 11	1
S 12	1
S 13	1
S 14	1
S 15	1
S 16	1
S 17	1
S 18	1
S 19	1
S 20	1
S 21	1
S 22	1
S 23	1
S 24	1
S 25	1
S 26	1
S 27	1

Table S2. Fingerprint clustering method classes

**Class ID**	**Class Size**
Class identifier. ID values of non-singleton classes generally have the form "C 1", "C 2", etc., while singleton classes have "S 1", "S 2", etc.	Number of compounds in the class.
C 1	47
C 2	21
C 3	21
C 4	17
C 5	9
C 6	8
C 7	7
C 8	7
C 9	6
C 10	6
C 11	6
C 12	5
C 13	5
C 14	5
C 15	5
C 16	5
C 17	4
C 18	4
C 19	4
C 20	4
C 21	3
C 22	3
C 23	3
C 24	3
C 25	3
C 26	3
C 27	2
C 28	2
C 29	2
C 30	2
C 31	2
C 32	2
C 33	2
C 34	2
S 1	1
S 2	1
S 3	1
S 4	1
S 5	1
S 6	1
S 7	1
S 8	1
S 9	1
S 10	1
S 11	1
S 12	1
S 13	1

S 14	1
S 15	1
S 16	1
S 17	1
S 18	1
S 19	1
S 20	1
S 21	1
S 22	1
S 23	1
S 24	1
S 25	1
S 26	1
S 27	1
S 28	1
S 29	1
S 30	1
S 31	1
S 32	1
S 33	1
S 34	1
S 35	1
S 36	1
S 37	1
S 38	1
S 39	1
S 40	1
S 41	1

Table S3. Error metrics for the IV route classified by the protein binding category

SIMID	Protein binding category	RMSE	R2_LIN	R2_LOG	SPEAR_LIN	SPEAR_LOG	RMSLE	afe	aafe	CCC_LOG	CCC_lin	two_fold	three_fold	ten_fold	n	PARAMETER
1) Direct Scaling Heps	High	20200	0.0966	0.392	0.584	0.584	20200	0.25	4.21	0.352	0.0519	24	42	86	50	AUC
1) Direct Scaling Heps	Moderate	3390	0.219	0.499	0.677	0.677	3390	0.805	1.86	0.686	0.373	62	80.9	99.2	382	AUC
2) Dilution Method Heps	High	19800	0.106	0.35	0.556	0.556	19800	1.31	2.53	0.578	0.312	44	60	98	50	AUC
2) Dilution Method Heps	Moderate	5450	0.454	0.491	0.66	0.66	5450	2.31	2.54	0.525	0.516	41.1	63.6	95.8	382	AUC
3) Unbound Heps	High	3.99E+05	0.404	0.573	0.713	0.713	3.99E+05	15.8	15.8	0.227	0.0551	4	4	28	50	AUC
3) Unbound Heps	Moderate	17300	0.43	0.374	0.542	0.542	17300	4.02	4.12	0.331	0.222	25.4	42.9	85.6	382	AUC
4) Back Calculated	High	2130	0.988	0.969	0.974	0.974	2130	1.01	1.15	0.984	0.994	100	100	100	50	AUC
4) Back Calculated	Moderate	1110	0.916	0.971	0.982	0.982	1110	0.998	1.13	0.984	0.951	98.7	100	100	382	AUC
5) Machine Learning	High	1.8E+04	0.131	0.27	0.465	0.465	1.8E+04	1.3	2.47	0.463	0.265	46	70	96	50	AUC
5) Machine Learning	Moderate	5E+03	0.144	0.295	0.476	0.476	5E+03	2.24	2.8	0.394	0.334	34.8	59.4	94	382	AUC
6) Austin Method Heps	High	3.26E+05	0.356	0.577	0.713	0.713	3.26E+05	8.85	9.17	0.323	0.0642	4	12	50	50	AUC
6) Austin Method Heps	Moderate	10900	0.462	0.435	0.599	0.599	10900	2.92	3.06	0.436	0.335	37.2	56.3	91.6	382	AUC

**1) Direct Scaling Heps**	High	23.6	0.137	0.221	0.424	0.424	23.6	3.99	4.19	0.21	0.153	24	42	86	50	CL
**1) Direct Scaling Heps**	Moderate	16.4	0.223	0.27	0.521	0.521	16.4	1.24	1.86	0.475	0.448	62	80.9	99.2	382	CL
**2) Dilution Method Heps**	High	9.82	0.0536	0.208	0.422	0.422	9.82	0.76	2.5	0.441	0.212	44	60	98	50	CL
**2) Dilution Method Heps**	Moderate	21.1	0.193	0.318	0.532	0.532	21.1	0.434	2.53	0.378	0.211	41.4	63.4	95.8	382	CL
**3) Unbound Heps**	High	12	0.0454	0.47	0.641	0.641	12	0.0633	15.8	0.169	0.0197	4	4	28	50	CL
**3) Unbound Heps**	Moderate	23.8	0.143	0.245	0.446	0.446	23.8	0.249	4.12	0.233	0.136	24.9	43.5	85.3	382	CL
**4) Back Calculated**	High	1.89	0.956	0.959	0.969	0.969	1.89	0.987	1.15	0.979	0.975	100	100	100	50	CL
**4) Back Calculated**	Moderate	4.05	0.948	0.958	0.978	0.978	4.05	1	1.13	0.978	0.973	98.7	100	100	382	CL
**5) Machine Learning**	High	9.38	0.0655	0.0749	0.361	0.361	9.38	0.767	2.48	0.21	0.12	42	70	96	50	CL
**5) Machine Learning**	Moderate	23.4	0.0144	0.0298	0.14	0.14	23.4	0.447	2.8	0.0904	0.0305	35.1	58.9	94	382	CL
**6) Austin Method Heps**	High	11.6	0.0343	0.466	0.626	0.626	11.6	0.113	9.19	0.244	0.0355	4	12	50	50	CL
**6) Austin Method Heps**	Moderate	22.1	0.177	0.295	0.507	0.507	22.1	0.343	3.06	0.321	0.189	37.2	56	91.6	382	CL
**1) Direct Scaling Heps**	High	2.3	0.0385	0.0967	0.29	0.29	2.3	0.85	2.31	0.297	0.176	61.2	73.5	89.8	49	VSS
**1) Direct Scaling Heps**	Moderate	2.84	0.475	0.441	0.648	0.648	2.84	0.674	1.97	0.6	0.645	58.8	82.6	98.1	374	VSS

**2) Dilution Method Heps**	High	2.37	0.0367	0.0969	0.296	0.296	2.37	0.866	2.32	0.297	0.168	59.2	73.5	89.8	49	VSS
**2) Dilution Method Heps**	Moderate	2.84	0.475	0.441	0.648	0.648	2.84	0.683	1.97	0.603	0.65	60.2	83.2	98.1	374	VSS
**3) Unbound Heps**	High	2.4	0.0356	0.0962	0.292	0.292	2.4	0.872	2.32	0.296	0.164	61.2	73.5	89.8	49	VSS
**3) Unbound Heps**	Moderate	2.84	0.474	0.441	0.648	0.648	2.84	0.685	1.96	0.604	0.651	60.7	83.4	98.1	374	VSS
**4) Back Calculated**	High	2.37	0.0348	0.0941	0.293	0.293	2.37	0.865	2.32	0.293	0.164	61.2	73.5	89.8	49	VSS
**4) Back Calculated**	Moderate	2.9	0.453	0.434	0.643	0.643	2.9	0.674	1.98	0.595	0.626	59.6	82.4	97.9	374	VSS
**5) Machine Learning**	High	4.61	0.00851	0.0175	0.303	0.303	4.61	3.09	4.54	0.077	0.0362	16.3	32.7	81.6	49	VSS
**5) Machine Learning**	Moderate	3.08	0.376	0.279	0.566	0.566	3.08	0.877	2.26	0.501	0.598	49.2	75.4	96.3	374	VSS
**6) Austin Method Heps**	High	2.4	0.0357	0.0963	0.292	0.292	2.4	0.871	2.32	0.296	0.165	61.2	73.5	89.8	49	VSS
**6) Austin Method Heps**	Moderate	2.83	0.475	0.442	0.648	0.648	2.83	0.684	1.96	0.603	0.65	60.4	83.2	98.1	374	VSS

Table S4. Error metrics for the IV route classified by the clearance category

SIMID	CL_CATEGORY	RMSE	R2_LIN	R2_LOG	SPEAR_LIN	SPEAR_LOG	RMSLE	afe	aafe	CCC_LOG	CCC_lin	two_fold	three_fold	ten_fold	n	PARAMETER
**1) Direct Scaling Heps**	High	661	0.445	0.52	0.701	0.701	661	1.69	1.72	0.542	0.459	67.3	85.7	100	49	AUC
**1) Direct Scaling Heps**	Moderate	1900	0.369	0.669	0.821	0.821	1900	1.08	1.47	0.813	0.525	82.5	91.2	100	160	AUC
**1) Direct Scaling Heps**	Low	1890	0.197	0.397	0.575	0.575	1890	0.558	2.19	0.49	0.387	48.3	74.1	99.3	143	AUC
**1) Direct Scaling Heps**	Very Low	17200	0.0943	0.584	0.697	0.697	17200	0.262	3.89	0.481	0.0642	18.8	45	88.8	80	AUC
**2) Dilution Method Heps**	High	3530	0.0373	0.17	0.402	0.402	3530	4.42	4.42	0.131	0.032	14.3	32.7	81.6	49	AUC
**2) Dilution Method Heps**	Moderate	5610	0.341	0.523	0.707	0.707	5610	2.88	2.94	0.426	0.214	31.9	55	95.6	160	AUC
**2) Dilution Method Heps**	Low	3950	0.198	0.328	0.542	0.542	3950	1.77	2.05	0.448	0.263	53.1	72	100	143	AUC
**2) Dilution Method Heps**	Very Low	1.7E+04	0.132	0.609	0.748	0.748	1.7E+04	1.12	1.95	0.774	0.357	56.2	82.5	98.8	80	AUC
**3) Unbound Heps**	High	10800	0.00491	0.0736	0.295	0.295	10800	7.97	7.97	0.0627	0.00435	6.12	18.4	63.3	49	AUC
**3) Unbound Heps**	Moderate	16500	0.149	0.335	0.533	0.533	16500	5.21	5.27	0.238	0.054	16.9	32.5	80	160	AUC
**3) Unbound Heps**	Low	17200	0.0111	0.148	0.407	0.407	17200	3.78	3.82	0.164	0.0176	32.2	46.9	84.6	143	AUC
**3) Unbound Heps**	Very Low	3.16E+05	0.369	0.568	0.703	0.703	3.16E+05	4.12	4.44	0.492	0.0564	28.7	47.5	76.2	80	AUC
**4) Back Calculated**	High	92.9	0.953	0.952	0.969	0.969	92.9	1.01	1.11	0.975	0.975	100	100	100	49	AUC
**4) Back Calculated**	Moderate	538	0.857	0.97	0.985	0.985	538	1.01	1.1	0.984	0.912	99.4	100	100	160	AUC
**4) Back Calculated**	Low	563	0.893	0.907	0.914	0.914	563	1	1.17	0.949	0.945	97.9	100	100	143	AUC
**4) Back Calculated**	Very Low	2760	0.971	0.969	0.977	0.977	2760	0.964	1.16	0.984	0.984	98.8	100	100	80	AUC
**5) Machine Learning**	High	4960	0.345	0.388	0.597	0.597	4960	6.55	6.55	0.116	0.0674	0	8.16	73.5	49	AUC
**5) Machine Learning**	Moderate	5480	0.473	0.582	0.736	0.736	5480	3.46	3.5	0.379	0.235	15.6	40.6	93.8	160	AUC
**5) Machine Learning**	Low	2820	0.294	0.41	0.55	0.55	2820	1.57	1.85	0.535	0.404	62.2	89.5	100	143	AUC
**5) Machine Learning**	Very Low	15300	0.153	0.604	0.678	0.678	15300	0.648	2.06	0.724	0.306	52.5	81.2	97.5	80	AUC
**6) Austin Method Heps**	High	6540	0.00794	0.103	0.355	0.355	6540	5.62	5.62	0.0923	0.00891	14.3	28.6	77.6	49	AUC
**6) Austin Method Heps**	Moderate	10200	0.202	0.412	0.611	0.611	10200	3.77	3.83	0.324	0.0989	25	46.9	85.6	160	AUC
**6) Austin Method Heps**	Low	8270	0.0468	0.203	0.432	0.432	8270	2.63	2.74	0.258	0.069	46.9	62.2	91.6	143	AUC
**6) Austin Method Heps**	Very Low	2.58E+05	0.324	0.625	0.735	0.735	2.58E+05	2.8	3.29	0.603	0.065	37.5	53.8	86.2	80	AUC
**1) Direct Scaling Heps**	High	27.2	3.32E-05	2.32E-04	0.103	0.103	27.2	0.593	1.72	0.00588	0.00247	67.3	85.7	100	49	CL
**1) Direct Scaling Heps**	Moderate	12.4	0.144	0.184	0.397	0.397	12.4	0.923	1.48	0.382	0.378	83.1	91.2	100	160	CL
**1) Direct Scaling Heps**	Low	17.9	0.00877	0.0018	0.0936	0.0936	17.9	1.79	2.18	0.0238	0.0388	47.6	74.1	99.3	143	CL

**1) Direct Scaling Heps**	Very Low	17.1	0.0581	0.0556	0.25	0.25	17.1	3.82	3.89	0.0811	0.0387	18.8	45	88.8	80	CL
**2) Dilution Method Heps**	High	43.2	0.0188	0.0138	-0.081	-0.081	43.2	0.227	4.41	-0.0143	-0.0174	14.3	32.7	83.7	49	CL
**2) Dilution Method Heps**	Moderate	21.3	0.0682	0.126	0.329	0.329	21.3	0.347	2.94	0.104	0.0913	31.9	54.4	95	160	CL
**2) Dilution Method Heps**	Low	8.11	0.0315	0.0337	0.193	0.193	8.11	0.565	2.05	0.106	0.13	53.8	72	100	143	CL
**2) Dilution Method Heps**	Very Low	4.74	0.0763	0.172	0.479	0.479	4.74	0.891	1.95	0.398	0.195	56.2	82.5	98.8	80	CL
**3) Unbound Heps**	High	47.8	0.0263	0.0144	-0.146	-0.146	47.8	0.126	7.94	-0.0104	-0.016	6.12	20.4	63.3	49	CL
**3) Unbound Heps**	Moderate	24.7	0.0505	0.0693	0.299	0.299	24.7	0.192	5.27	0.049	0.0593	16.2	32.5	79.4	160	CL
**3) Unbound Heps**	Low	9.95	0.0571	0.051	0.24	0.24	9.95	0.264	3.83	0.0609	0.0703	30.8	47.6	84.6	143	CL
**3) Unbound Heps**	Very Low	3.11	0.209	0.371	0.546	0.546	3.11	0.243	4.43	0.262	0.294	30	47.5	76.2	80	CL
**4) Back Calculated**	High	7.85	0.637	0.643	0.793	0.793	7.85	0.997	1.1	0.792	0.782	100	100	100	49	CL
**4) Back Calculated**	Moderate	3.91	0.871	0.841	0.919	0.919	3.91	0.988	1.1	0.916	0.931	99.4	100	100	160	CL
**4) Back Calculated**	Low	2.51	0.78	0.705	0.837	0.837	2.51	0.994	1.17	0.833	0.877	97.9	100	100	143	CL
**4) Back Calculated**	Very Low	0.877	0.819	0.9	0.909	0.909	0.877	1.04	1.16	0.947	0.903	98.8	100	100	80	CL
**5) Machine Learning**	High	48.1	0.0332	0.0329	-0.0821	-0.0821	48.1	0.153	6.52	-0.0101	-0.00858	0	8.16	73.5	49	CL
**5) Machine Learning**	Moderate	23.9	0.00439	0.002	0.11	0.11	23.9	0.289	3.5	0.00837	0.0117	15.6	39.4	93.8	160	CL
**5) Machine Learning**	Low	7.49	0.00798	0.0111	0.0806	0.0806	7.49	0.635	1.85	0.0689	0.054	62.2	89.5	100	143	CL
**5) Machine Learning**	Very Low	3.96	0.0567	0.0563	0.264	0.264	3.96	1.54	2.07	0.192	0.165	51.2	81.2	97.5	80	CL
**6) Austin Method Heps**	High	45	0.0178	0.00436	-0.084	-0.084	45	0.179	5.6	-0.00731	-0.0164	12.2	28.6	77.6	49	CL
**6) Austin Method Heps**	Moderate	22.8	0.0706	0.114	0.339	0.339	22.8	0.265	3.83	0.0808	0.088	25.6	46.2	85.6	160	CL
**6) Austin Method Heps**	Low	8.75	0.0887	0.0729	0.311	0.31	8.75	0.38	2.74	0.102	0.147	46.9	62.2	91.6	143	CL
**6) Austin Method Heps**	Very Low	3.48	0.163	0.408	0.565	0.565	3.48	0.357	3.29	0.35	0.314	37.5	53.8	86.2	80	CL
**1) Direct Scaling Heps**	High	4.72	0.692	0.545	0.676	0.676	4.72	0.442	2.46	0.545	0.571	42.9	75.5	95.9	49	VSS
**1) Direct Scaling Heps**	Moderate	2.74	0.612	0.454	0.634	0.634	2.74	0.563	2.06	0.545	0.683	56.6	79.9	96.9	159	VSS
**1) Direct Scaling Heps**	Low	2.13	0.506	0.424	0.645	0.645	2.13	0.843	1.91	0.612	0.68	63.8	83.3	98.6	138	VSS
**1) Direct Scaling Heps**	Very Low	2.19	0.0765	0.3	0.547	0.547	2.19	0.991	1.84	0.529	0.213	66.2	85.7	96.1	77	VSS
**2) Dilution Method Heps**	High	4.68	0.685	0.543	0.675	0.675	4.68	0.45	2.43	0.55	0.579	42.9	77.6	95.9	49	VSS
**2) Dilution Method Heps**	Moderate	2.71	0.61	0.453	0.636	0.636	2.71	0.571	2.05	0.549	0.692	58.5	80.5	96.9	159	VSS
**2) Dilution Method Heps**	Low	2.18	0.501	0.422	0.642	0.642	2.18	0.855	1.91	0.612	0.673	63.8	83.3	98.6	138	VSS
**2) Dilution Method Heps**	Very Low	2.24	0.0726	0.296	0.547	0.547	2.24	1	1.85	0.524	0.204	67.5	85.7	96.1	77	VSS
**3) Unbound Heps**	High	4.67	0.683	0.542	0.675	0.675	4.67	0.452	2.42	0.551	0.581	46.9	77.6	95.9	49	VSS
**3) Unbound Heps**	Moderate	2.71	0.61	0.453	0.636	0.636	2.71	0.573	2.04	0.55	0.693	58.5	81.1	96.9	159	VSS

**3) Unbound Heps**	Low	2.19	0.499	0.421	0.641	0.641	2.19	0.858	1.91	0.612	0.671	64.5	83.3	98.6	138	VSS
**3) Unbound Heps**	Very Low	2.26	0.0712	0.294	0.545	0.545	2.26	1	1.85	0.523	0.201	67.5	85.7	96.1	77	VSS
**4) Back Calculated**	High	4.9	0.677	0.536	0.675	0.675	4.9	0.432	2.5	0.533	0.534	42.9	75.5	95.9	49	VSS
**4) Back Calculated**	Moderate	2.76	0.607	0.45	0.634	0.634	2.76	0.562	2.06	0.543	0.673	57.9	79.9	96.2	159	VSS
**4) Back Calculated**	Low	2.16	0.497	0.419	0.642	0.642	2.16	0.851	1.91	0.61	0.674	63.8	82.6	98.6	138	VSS
**4) Back Calculated**	Very Low	2.25	0.0705	0.294	0.547	0.547	2.25	1	1.85	0.522	0.201	67.5	85.7	96.1	77	VSS
**5) Machine Learning**	High	4.75	0.54	0.38	0.641	0.641	4.75	0.542	2.36	0.508	0.547	51	73.5	95.9	49	VSS
**5) Machine Learning**	Moderate	2.89	0.475	0.351	0.63	0.63	2.89	0.683	2.27	0.504	0.661	50.3	76.1	94.3	159	VSS
**5) Machine Learning**	Low	2.77	0.398	0.241	0.557	0.557	2.77	1.31	2.37	0.452	0.538	44.2	70.3	97.1	138	VSS
**5) Machine Learning**	Very Low	3.78	0.0242	0.0638	0.203	0.203	3.78	2.16	3.09	0.178	0.0672	33.8	57.1	89.6	77	VSS
**6) Austin Method Heps**	High	4.68	0.685	0.543	0.675	0.675	4.68	0.451	2.43	0.55	0.579	44.9	77.6	95.9	49	VSS
**6) Austin Method Heps**	Moderate	2.71	0.61	0.453	0.637	0.637	2.71	0.572	2.05	0.55	0.692	58.5	80.5	96.9	159	VSS
**6) Austin Method Heps**	Low	2.19	0.498	0.421	0.641	0.641	2.19	0.857	1.91	0.612	0.671	64.5	83.3	98.6	138	VSS
**6) Austin Method Heps**	Very Low	2.25	0.0723	0.295	0.545	0.545	2.25	1	1.85	0.523	0.203	67.5	85.7	96.1	77	VSS

Table S5. Full error metrics for the IV route

SIMID	RMSE	R2_LIN	R2_LOG	SPEAR_LIN	SPEAR_LOG	RMSLE	afe	aafe	CCC_LOG	CCC_lin	two_fold	three_fold	ten_fold	n	PARAMETER
1) Direct Scaling Heps	7580	0.0782	0.419	0.622	0.622	7580	0.703	2.05	0.603	0.128	57.6	76.4	97.7	432	AUC
2) Dilution Method Heps	8470	0.216	0.471	0.638	0.638	8470	2.16	2.53	0.545	0.44	41.4	63.2	96.1	432	AUC
3) Unbound Heps	1.37E+05	0.401	0.436	0.564	0.564	1.37E+05	4.71	4.81	0.364	0.0679	22.9	38.4	78.9	432	AUC
4) Back Calculated	1270	0.974	0.972	0.982	0.982	1270	1	1.14	0.986	0.986	98.8	100	100	432	AUC
5) Machine Learning	7720	0.129	0.308	0.489	0.489	7720	2.1	2.76	0.422	0.323	36.1	60.6	94.2	432	AUC
6) Austin Method Heps	1.12E+05	0.353	0.489	0.611	0.611	1.12E+05	3.31	3.48	0.464	0.0779	33.3	51.2	86.8	432	AUC
1) Direct Scaling Heps	17.4	0.179	0.222	0.471	0.471	17.4	1.42	2.05	0.398	0.397	57.6	76.4	97.7	432	CL
2) Dilution Method Heps	20.1	0.198	0.33	0.541	0.541	20.1	0.463	2.53	0.423	0.227	41.7	63	96.1	432	CL
3) Unbound Heps	22.8	0.181	0.379	0.528	0.528	22.8	0.212	4.81	0.309	0.159	22.5	38.9	78.7	432	CL
4) Back Calculated	3.86	0.952	0.964	0.98	0.98	3.86	1	1.13	0.981	0.975	98.8	100	100	432	CL
5) Machine Learning	22.2	0.0391	0.0902	0.246	0.246	22.2	0.476	2.76	0.176	0.0546	35.9	60.2	94.2	432	CL
6) Austin Method Heps	21.2	0.217	0.419	0.574	0.574	21.2	0.302	3.48	0.397	0.218	33.3	50.9	86.8	432	CL
1) Direct Scaling Heps	2.78	0.449	0.401	0.603	0.603	2.78	0.692	2.01	0.582	0.634	59.1	81.6	97.2	423	VSS
2) Dilution Method Heps	2.79	0.447	0.4	0.602	0.602	2.79	0.702	2	0.584	0.638	60	82	97.2	423	VSS
3) Unbound Heps	2.79	0.446	0.399	0.602	0.602	2.79	0.704	2	0.584	0.638	60.8	82.3	97.2	423	VSS
4) Back Calculated	2.85	0.425	0.392	0.598	0.598	2.85	0.694	2.02	0.576	0.615	59.8	81.3	96.9	423	VSS
5) Machine Learning	3.3	0.29	0.182	0.46	0.46	3.3	1.01	2.45	0.412	0.527	45.4	70.4	94.6	423	VSS
6) Austin Method Heps	2.79	0.447	0.399	0.602	0.602	2.79	0.703	2	0.584	0.637	60.5	82	97.2	423	VSS

Table S6. Full error metrics for the oral route

SIMID	RMSE	R2_LIN	R2_LOG	SPEAR_LIN	SPEAR_LOG	RMSLE	afe	aafe	CCC_LOG	CCC_lin	two_fold	three_fold	ten_fold	n	PARAMETER
1) Direct Scaling Heps	41800	0.0755	0.367	0.6	0.6	41800	0.589	3.29	0.559	0.11	38	56.8	86.2	479	AUC
2) Dilution Method Heps	37600	0.254	0.473	0.673	0.673	37600	2.62	3.57	0.55	0.475	31.9	50.4	87.5	480	AUC
3) Austin Method Heps	3.54E+05	0.229	0.477	0.662	0.662	3.54E+05	4.13	4.8	0.502	0.105	23.3	40.8	78.8	480	AUC
4) Back Calculated CL + in vitro	33700	0.384	0.654	0.855	0.855	33700	0.79	2.12	0.801	0.511	59.4	80	96.2	480	AUC

**5) ML Physchem + Back Calculated CL**	30500	0.497	0.682	0.858	0.858	30500	0.905	2.01	0.825	0.688	63.5	81.9	96.2	480	AUC
**6) ML All properties**	31100	0.475	0.322	0.512	0.512	31100	2.9	4.2	0.417	0.668	27.9	45.4	81.7	480	AUC
**7) HT-PBPK**	31900	0.456	0.686	0.869	0.869	31900	0.752	2.05	0.818	0.576	63.1	80.6	96	480	AUC
**1) Direct Scaling Heps**	3730	0.111	0.32	0.561	0.561	3730	0.884	2.97	0.563	0.315	40.5	58	91	479	CMAX
**2) Dilution Method Heps**	4970	0.206	0.395	0.618	0.618	4970	2.13	3.12	0.549	0.409	38.8	59	87.1	480	CMAX
**3) Austin Method Heps**	6420	0.273	0.408	0.622	0.622	6420	2.51	3.34	0.532	0.396	36.9	54.6	85.6	480	CMAX
**4) Back Calculated CL + in vitro**	3270	0.359	0.514	0.755	0.755	3270	1.03	2.46	0.713	0.599	47.5	72.5	95.4	480	CMAX
**5) ML Physchem + Back Calculated CL**	4470	0.447	0.555	0.758	0.758	4470	1.53	2.53	0.715	0.587	48.1	66.2	94.4	480	CMAX
**6) ML All properties**	6550	0.133	0.289	0.531	0.531	6550	2.41	3.69	0.453	0.289	33.5	50.4	81.9	480	CMAX
**7) HT-PBPK**	4370	0.412	0.54	0.752	0.752	4370	1.35	2.45	0.716	0.577	49.4	70.8	94	480	CMAX
**1) Direct Scaling Heps**	107	0.0227	0.0477	0.307	0.307	107	0.833	1.89	0.203	0.0607	66.3	84.9	97.3	410	FORAL
**2) Dilution Method Heps**	105	0.0161	0.0218	0.257	0.257	105	1.22	1.85	0.123	0.0515	68.6	85.4	96.8	411	FORAL
**3) Austin Method Heps**	106	0.0142	0.018	0.221	0.221	106	1.26	1.88	0.109	0.0491	68.6	85.2	96.8	411	FORAL
**4) Back Calculated CL + in vitro**	107	0.0241	0.0547	0.309	0.309	107	0.808	2.05	0.222	0.0724	64.5	83	95.9	411	FORAL
**5) ML Physchem + Back Calculated CL**	106	0.0253	0.053	0.307	0.307	106	0.928	1.95	0.227	0.0734	68.1	84.7	96.1	411	FORAL
**6) ML All properties**	107	0.00425	0.0016	0.157	0.157	107	1.46	1.94	0.0238	0.0205	65.9	82.7	97.3	411	FORAL
**7) HT-PBPK**	106	0.0226	0.0566	0.327	0.327	106	0.862	1.96	0.23	0.0687	65.9	83.5	95.9	411	FORAL
